# Inhibitor of DNA Binding 4 (ID4) Is Highly Expressed in Human Melanoma Tissues and May Function to Restrict Normal Differentiation of Melanoma Cells

**DOI:** 10.1371/journal.pone.0116839

**Published:** 2015-02-02

**Authors:** Yuval Peretz, Hong Wu, Shayan Patel, Alfonso Bellacosa, Richard A. Katz

**Affiliations:** Fox Chase Cancer Center, Temple University Health System, Philadelphia, Pennsylvania, United States of America; University of Minnesota Medical School, UNITED STATES

## Abstract

Melanoma tissues and cell lines are heterogeneous, and include cells with invasive, proliferative, stem cell-like, and differentiated properties. Such heterogeneity likely contributes to the aggressiveness of the disease and resistance to therapy. One model suggests that heterogeneity arises from rare cancer stem cells (CSCs) that produce distinct cancer cell lineages. Another model suggests that heterogeneity arises through reversible cellular plasticity, or phenotype-switching. Recent work indicates that phenotype-switching may include the ability of cancer cells to dedifferentiate to a stem cell-like state. We set out to investigate the phenotype-switching capabilities of melanoma cells, and used unbiased methods to identify genes that may control such switching. We developed a system to reversibly synchronize melanoma cells between 2D-monolayer and 3D-stem cell-like growth states. Melanoma cells maintained in the stem cell-like state showed a striking upregulation of a gene set related to development and neural stem cell biology, which included SRY-box 2 (SOX2) and Inhibitor of DNA Binding 4 (ID4). A gene set related to cancer cell motility and invasiveness was concomitantly downregulated. Intense and pervasive ID4 protein expression was detected in human melanoma tissue samples, suggesting disease relevance for this protein. SiRNA knockdown of ID4 inhibited switching from monolayer to 3D-stem cell-like growth, and instead promoted switching to a highly differentiated, neuronal-like morphology. We suggest that ID4 is upregulated in melanoma as part of a stem cell-like program that facilitates further adaptive plasticity. ID4 may contribute to disease by preventing stem cell-like melanoma cells from progressing to a normal differentiated state. This interpretation is guided by the known role of ID4 as a differentiation inhibitor during normal development. The melanoma stem cell-like state may be protected by factors such as ID4, thereby potentially identifying a new therapeutic vulnerability to drive differentiation to the normal cell phenotype.

## Introduction

Malignant melanoma is a potentially deadly type of skin cancer that occurs as a result of melanocyte transformation [1]. Although melanoma is relatively rare, it has become a major concern due to an increased incidence over the past two decades. In the early stages, melanoma is generally curable with surgical intervention, yet once metastasized to organ sites, the prognosis becomes very poor. As melanoma is highly resistant to many conventional therapies, there is an urgent need for new diagnostic, prognostic, and treatment approaches. The genetic lesions in melanoma, including NRAS and BRAF mutations, are well characterized, and activated BRAF kinase has been demonstrated to be an effective drug target for melanoma therapy [2]. However, acquired drug resistance to this class of inhibitor has been described [3]. Beyond targeting genetic lesions, an immunomodulatory approach for melanoma treatment has recently shown exciting promise [4].

Cellular phenotypic heterogeneity underlies difficulties in melanoma diagnoses and treatment, and may impact the aforementioned emerging therapies [5–7]. The mechanisms by which such heterogeneity arises in melanoma are the subject of intense research. Over the past decade, the cancer stem cell (CSC) model has emerged in relation to the basic nature of cancer, as well as to explain tumor heterogeneity. The model states that CSCs function to initiate and sustain heterogeneous tumors through hierarchical cell division processes reminiscent of normal stem cell differentiation [8]. The main features of the model are that CSCs represent only a small fraction of tumor cells (ca. 0.5% to 5.0%), have an endless capacity to self-renew, and are fully responsible for the growth of tumors. Significant support for the CSC model has emerged [9]. However, there is a controversy as to whether melanoma follows the CSC model [10–16]. Recent studies have indicated that melanoma tumors are highly enriched (> 20%) with cells capable of initiating and maintaining heterogeneous tumors, a fraction that is inconsistent with the CSC model [13,14]. The term “tumor-initiating cells” (TICs) [17] has been used to more accurately describe cells capable of forming heterogeneous tumors, without any assumptions as to whether the cells display the hallmarks of stem cells, such as self-renewal and asymmetric division. To explain tumor heterogeneity in the absence of a hierarchical CSC model, it has been proposed that heterogeneous melanoma tumors can be formed from single TICs through epigenetic processes: reversible phenotypic plasticity, or phenotype-switching [7,13–16].

A further complication of the models for melanoma heterogeneity is that melanoma tumors can display pervasive stem cell-factor expression, and “stem cell-like” cells can be detected [13,18–20]. These findings raise questions regarding the function of these stem cell-like cells in melanoma, if not as CSCs. One explanation for the presence of stem cell-like cells is that the melanoma disease process exploits the developmental program [18,21]. Alternatively, melanoma cells may be able to acquire stem cell-like properties in order to provide an intermediate state for regenerating heterogeneity [22,23]. Epigenetic plasticity of melanoma cells, including acquisition of stem cell-like behavior, can indeed be triggered by the cellular microenvironment, potentially providing extraordinary adaptability [21,24,25].

Based on our interest in epigenetic networks [26], we sought to develop a tissue culture system to identify regulatory factors that might play a functional role in epigenetic-based, reversible phenotype-switching between the proliferative melanoma cancer cell state and the stem cell-like state. To this end, we have adapted features of the melanoma 3-dimensional (3D) spheroid cell culture system [27]. Spheroids have been explored as models for tumor mimicry, tumorgenic capacity, heterogeneity, drug resistance, and differentiation capability of cancer cells [28]. Furthermore, CSCs are frequently propagated as spheroids. We developed an experimental strategy to monitor reversible transitions from proliferative melanoma cell monolayer growth to stem cell-like 3D-spheroid growth. By comparing the transcriptomes of cells grown under these two conditions, we identified an upregulated neural progenitor gene expression profile in spheroids that included Inhibitor of DNA Binding 4 (ID4), a transcription factor. Here we show that high levels of ID4 protein are present in human melanoma tissue samples and siRNA-based knockdown of ID4 in cultured melanoma cells promoted progression to a highly differentiated, more normal cell state. Our findings support a model whereby melanoma stem cell-like cells can be pervasive, and serve as advantageous plastic intermediates, possibly for generating heterogeneous tumor cells; furthermore, the stem cell-like state must be protected by factors such as ID4 that prevent normal differentiation.

## Materials and Methods

### Ethics Statement

The study was reviewed by the Fox Chase Cancer Center Institutional Review Board and received exempt status, IRB# 11–846.

### Cell Culture

MNT-1 cells [29] were provided by Michael Marks (University of Pennsylvania) and cultured in DMEM media supplemented with 20% Hyclone FCS (Thermo Scientific, Pittsburgh, PA), 10% AIM V (Life Technologies, Carlsbad, CA), sodium pyruvate, nonessential amino acids, and L-glutamine. The 1205Lu [30] and 451Lu [31] cells were obtained from Meenhard Herlyn (Wistar Institute), and C8161 [32] cells were obtained from Mary Hendrix (Northwestern University). SK-MEL-28 cells [33] were obtained by the Fox Chase Cancer Cell Culture Facility as part of the NCI 60 cell line panel. The1205Lu, 451Lu, C8161 and SK-MEL-28 cells were cultured in RPMI-1640 media supplemented with 10% FCS. For propagation under stem cell conditions, hESC media was prepared by supplementing DMEM/F-12 with 20% KnockOut Serum Replacement (cat. no. 10828-028, Life Technologies, Carlsbad, CA), nonessential amino acids, 0.5X L-glutamine and 0.1 mM 2-mercaptoethanol. Stemgent NutriStem XF/FF Culture Medium (cat. no. 01-0005) also promoted 1205Lu spheroid/MB formation.

### Immunocytochemistry

Cells were fixed in 4% paraformaldehyde in PBS for 15 minutes, and then washed with ice cold PBS. Cells membranes were permeabilized using 0.25% Triton X-100 (10 minutes), followed by washing with PBS. Samples were blocked with 1% BSA in PBS-Tween 20 for 60 minutes, and then incubated for 1 hour with primary antibody diluted (1:200–1:500) in DaVinci Green Diluent (cat. no. PD900, Biocare Medical, Concord, CA). The following primary antibodies were used: mouse anti-TUJ-1 (cat. no. sc-58888, Santa Cruz Biotechnology, Santa Cruz, CA), rabbit anti-Sox2 (cat. no. sc-20088, Santa Cruz Biotechnology), rabbit anti-ID4 (cat. no. sc-13047, Santa Cruz Biotechnology), rabbit anti-Notch1 (cat. no. 07-1232, EMD Millipore, Billerica, MA), mouse anti-Nestin (cat. no. MAB5326, EMD Millipore, Billerica, MA). Samples were then washed in PBS and incubated with Molecular Probes secondary antibodies (Life Technologies, Carlsbad, CA): Alexa Fluor 488 goat anti-rabbit (cat. no. A11001), Alexa Fluor 488 goat anti-mouse (cat. no. A11008), and Alexa Fluor 555 donkey anti-rabbit (cat. no. A31572). Secondary antibodies were diluted in DaVinci Green Diluent (1:500) and incubation was for 60 min. After final washing in PBS, images were captured using an Olympus IMT-2 microscope.

### Immunohistochemistry (IHC)

Tissue microarray (TMA) slides were prepared from anonymized samples procured at Fox Chase Cancer Center in accordance with the policies of the Fox Chase Cancer Center Internal Review Board. Slides were prepared using formalin-fixed paraffin-embedded samples. TMA slides were baked at 60°C for 1 hour, deparaffinized with xylene, and hydrated with graded ethanol to double-distilled water (ddH_2_0). Antigen was retrieved by immersing the slides in 0.1M citrate buffer, followed by boiling and washing in ddH_2_0. Endogenous peroxidase was quenched using 3% hydrogen peroxide (Sigma-Aldrich, St. Louis, MO) in methanol for 10 min. After washing in ddH_2_0, slides were placed in TBS-Tween 20 (TBST). Slides were then blocked in Background Sniper (cat. no. BS966, Biocare Medical, Concord, CA), followed by incubation with the primary anti-ID4 antibody (cat. no. sc-13047, Santa Cruz Biotechnology, Santa Cruz, CA) diluted in DaVinci Green Diluent (1: 500). After an overnight incubation at 4°C, slides were washed and then incubated with MACH 4 universal HRP-polymer detector (cat. no. M4U534, Biocare Medical, Concord, CA) for 30 min and then washed with TBST. Slides were then incubated with a DAB solution (cat. no. DB801, Biocare Medical, Concord, CA) for 2 min and washed with ddH_2_0. Slides were counterstained with CAT Hematoxylin (cat. no. CATHE, Biocare Medical, Concord, CA) for 30 sec and then washed with ddH_2_O. After dehydration with ethanol and clearing with xylenes, the slides were mounted with Permount (cat. no. S70104, Thermo Scientific, Pittsburgh, PA).

### SiRNA Knockdown Experiments

Cells were plated in a 96-well plate (1.5–2 × 10^4^ cells per well). The next day, an siRNA pool mix (i.e. a mixture of four independent siRNAs) was applied in a 100 μl OPTI-MEM media solution (cat. no. 31985, Life Technologies, Carlsbad, California) consisting of 100 nM siRNAs, plus 0.3 μl of Dharmafect 1 transfection reagent (cat. no. T-2001, Thermo Scientific, Pittsburgh, PA). For single siRNAs, a 25 nM to 50 nM final siRNA concentration was used. Cells were incubated with siRNAs for 48 hours, and then cultured in hESC or RPMI-1640 10% FCS media. All siRNAs were purchased from Qiagen, Valencia, CA.: ID4 (cat. no. Hs_ID4_4/5/6/7), Sox2 (cat. no. Hs_Sox2_4/5/6/7), Notch1 (cat. no. Hs_Notch1_1/2/3/4).

### Lentivirus Vector shRNA Infections

Lentiviral transduction particles (Mission) were purchased from Sigma-Aldrich, Saint Louis, MO. For targeting ID4 (shRNA vector cat. no. SHCLNV-NM_001546), the following individual shRNA particle preparations were used (Sh*ID4*#1 TRCN0000017323, Sh*ID4*#2 TRCN0000017326, Sh*ID4*#3 TRCN0000017327). Particles encoding a nonspecific shRNA (cat. no. SHC002V) were used as a control. For transduction, cells were plated in 48-well dishes (5 × 10^4^ cells per well) and were left to adhere overnight. Next, 2 × 10^6^ TU/ml viral particles were added to 200 μl of OPTI-MEM containing Hexadimethrine (cat. no. H9268, Sigma-Aldrich, Saint Louis, MO) to a final concentration of 5 μg/ml. Cells and viral particles were incubated for 6 hours at 37°C and the infection mix was replaced by OPTI-MEM for another 18 hours. Next, cells were cultured in RPMI-1640 supplemented with 10% FCS for four days, at which time puromycin was added at a final concentration of 1 μg/ml for selection of shRNA-expressing cells.

### Expression Microarray

Agilent SurePrint G3 Human Gene Expression slides were used (Agilent, Santa Clara, CA). This human genome array contains 44,000 printed 65mer oligos targeting 27,958 Entrez gene RNAs (4 × 44K). Total RNA was isolated using RNAqueous-4PCR Kit (cat. no. AM1914, Life Technologies, Carlsbad, California) according to the manufacturer’s protocol. Labeling and hybridization was carried out as follows. Briefly, 50–200 ng of total cellular RNA was amplified and labeled using the Agilent Low Input Quick Amp Labeling Kit (cat. no. 5190-2305) following the manufacturer’s protocol. Cy3-labeled cRNA (1.65 μg) was hybridized to the Agilent 4 × 44K Whole Genome Array for 17 hours at 65°C and washed according to manufacturer’s protocol. Array scanning and data extraction were performed as follows: The hybridized slides were scanned at 5μm resolution on the Agilent Microarray Scanner and fluorescent intensities of hybridization signals were extracted using the Agilent Feature Extraction software. For data analysis, statistics were performed using Microsoft Excel and MeV v4.7 TM4 open-source software [34]. Up- and down-regulated genes were defined by producing a five-fold difference in signal intensity in relation to an average of three housekeeping genes (see Results section). In order to exclude potential noise from low expressing genes, a second criterion was that the raw expression level was greater than 100 arbitrary units (a.u.). The complete expression microarray dataset was been deposited in the Gene Expression Omnibus (GEO) database repository, accession number GSE62849. Gene Ontology (GO) analysis was performed using g:Profiler (http://biit.cs.ut.ee/gprofiler/). Uncharacterized genes were not included in the analysis.

### Western Blot Analysis

Western blot analyses were performed using standard methods. The following primary antibodies were used: anti-Sox2 (cat. no. 2748; Cell Signaling, Danvers, MA), anti-Notch1 (cat. no. 07-1232; EMD Millipore, Billerica, MA), anti-ID4 (cat. no. sc-13047; Santa Cruz Biotechnology, Santa Cruz, CA), anti-GAPDH (cat. no. MAB374; EMD Millipore, Billerica, MA). Goat anti-rabbit (cat. no. 31462; Thermo Scientific, Pittsburgh, PA) and goat anti-mouse (cat. no. AP124P; EMD Millipore, Billerica, MA) were used as secondary antibodies. For detection, Pierce enhanced chemiluminescence reagents (Thermo Scientific, Pittsburgh, PA) were used according to the supplier’s protocols.

### Real-time PCR

Total RNA was isolated using RNAqueous-4PCR Kit (cat. no. AM1914, Life Technologies, Carlsbad, CA) according to the supplier’s protocol. The cDNA preparation was performed using Superscript II reverse transcriptase (cat. no. 18064-014, Life Technologies, Carlsbad, California) according to supplier’s protocol. Real-time PCR was performed using the Kapa SYBR FAST qPCR Kit (cat. no. KK4602, Kapa Biosystems, Woburn, MA) according to supplier’s protocol. The PCR protocol utilized 40 cycles with a two step program (95°C 5 sec, 60°C 30 sec). Reactions were performed using an Eppendorf Mastercycler (Eppendorf, Hauppauge, NY), and data were extracted and analyzed using the Eppendorf Realplex software.

### Stable Fluorescent Cell Lines

pDsRed2-Nuc and pEGFP vectors (Clontech Laboratories, Mountain View, CA) were used for stable transfection of 1205Lu cells. Cells were transfected using Lipofectamine 2000 (cat. no. 11668027, Life Technologies, Carlsbad, California). Expressing cells were selected with G418 and sorted for fluorescence.

## Results

### Assembly of Melanoma 1205Lu Cells Into Adherent 3D-Stem Cell-Like Bodies

**Figure 1:**
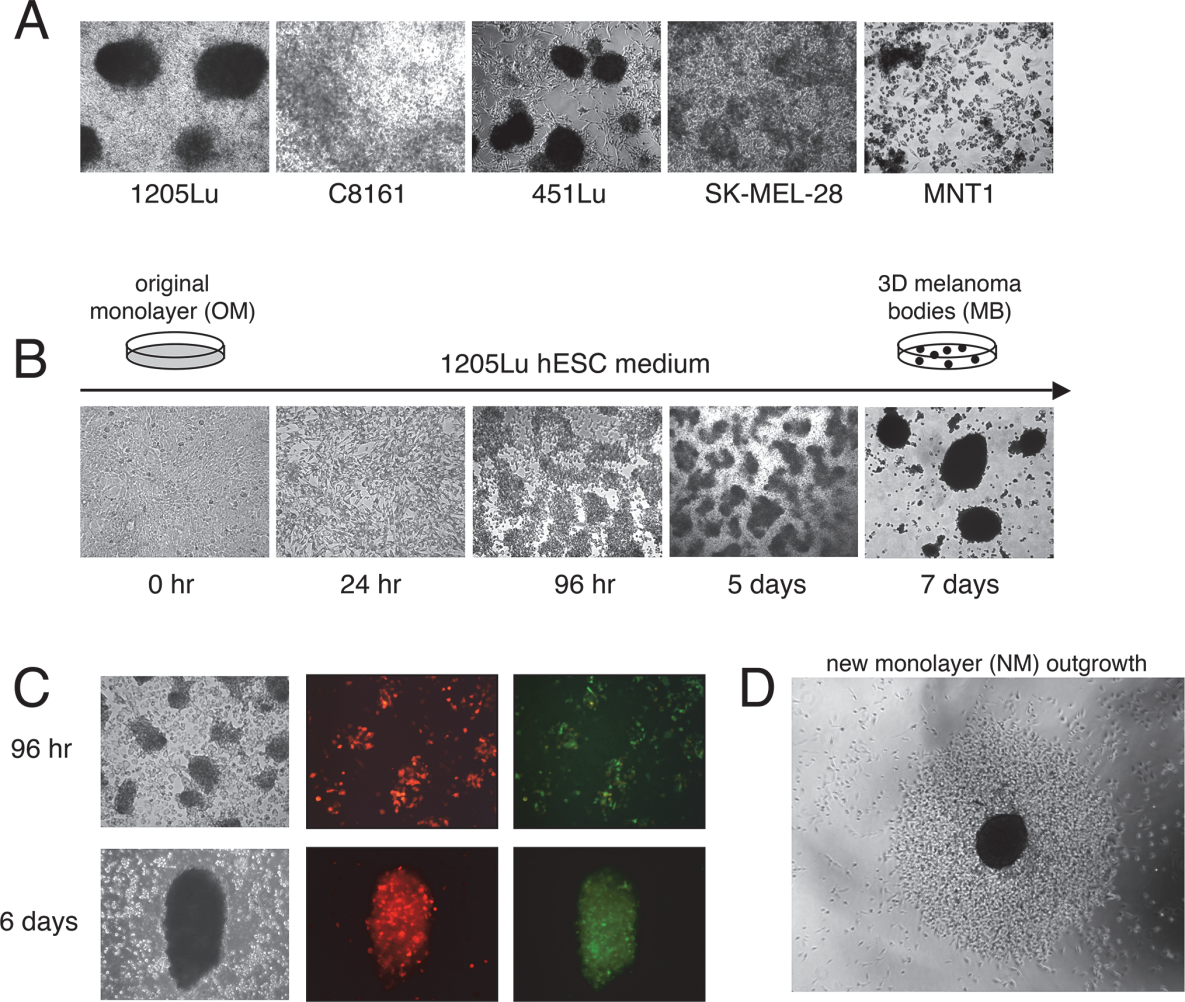
Formation of 3D-MBs under hESC conditions. (**A)** Survey of melanoma cell lines for the ability to form attached 3D-spheroids in hESC medium. The 1205Lu cell line formed attached 3D-spheroids, the 451Lu cell line formed floating spheroids, while the other tested cell lines displayed only various degrees of aggregation. **(B)** Formation of attached 1205Lu 3D-MBs after transfer to hESC medium over time. (**C)** To confirm that 1205Lu MBs formed by aggregation, 1205Lu GFP-labeled cells and 1205Lu nuclear dsRed-labeled were mixed and cultured as described in Panel A. **(D)** Mature 1205Lu 3D-MBs were removed by pipetting and were re-plated under standard monolayer media growth conditions, under which they reseeded a new monolayer. Shown is monolayer outgrowth after 96 hours.

To better understand processes underlying the phenotype-switching of melanoma cells, we adopted a 3D-culture system, as numerous studies have indicated that formation of melanoma 3D-spheroids enriches for stem cell-like growth [19,27,28,35–37]. To promote spheroid formation, melanoma cells are typically cultured under conditions that favor non-adherent cell growth (e.g. agar-coated plates or stem cell medium). To investigate potential reversible phenotype-switching between 2D-proliferative and 3D-stem cell like growth, we sought to derive conditions that favored formation of substrate-adherent 3D-melanoma spheroids. Such behavior would enable the study 3D-spheroid cells as they returned to monolayer growth, as described below in detail. Five human metastatic melanoma cell lines, 1205Lu, 451Lu, SK-MEL-28, C8161 and MNT1, were surveyed for their ability to form adherent 3D-spheroids when cultured in human embryonic stem cell (hESC) medium (Fig. 1A). Formation of spheroids from 1205Lu and 451Lu 2D-monolayer cells was observed in 5 to 7 days. The spheroids formed by 1205Lu cells were adherent, while those formed by 451Lu cells readily detached from the plate surface. For nomenclature to distinguish between floating versus attached spheroids, we refer to the attached 1205Lu spheroids as “melanoma bodies” (MBs). Various analyses indicated that the serum replacement component was essential for promoting 1205Lu MB formation (data not shown; see Materials and Methods).

As the formation of 3D-melanoma spheroids is believed to be a physical indicator of stem cell-like growth, we set out to use formation of these structures as a readout. This readout could then be used to identify factors that are required for specific steps in the transition from 2D to 3D-stem cell-like growth. The discrete morphological steps in 1205Lu MB formation are shown in Fig. 1B. Twenty-four hours after replacing standard medium with hESC medium, cell nuclei demonstrated a dramatic change, which was followed by formation of multi-cell structures within 96 hours. At seven days, well-organized 1205Lu 3D-MBs were observed (Fig. 1B). MB formation is initiated by cell aggregation (as opposed to clonal cell outgrowth), as demonstrated by mixing GFP-marked and nuclear dsRed-marked 1205Lu cells (Fig. 1C). Cell aggregates containing both red and green cells began to form at 96 hours after a shift to hESC medium, and mature red-green mixed MBs were observed.

Propagation of 1205Lu cells in hESC media and concomitant MB formation promoted slower growth with an increase in the G1/G0 fraction (S1 Fig.). Quiescence (G0) is a feature of adult neural stem cells, and lengthening of G1 is associated with differentiation [38]. In contrast, embryonic stem cells display very short Gap phases [39,40]. We suggest that the increase in G1/G0, as compared to the control, reflects an adult stem cell-like G0 state.

Similar to stem cell culture systems, daily replenishment with hESC medium was required to inhibit outgrowth of a new monolayer of proliferative melanoma cells. Under such media replenishment conditions, the MBs could remain attached and compact for months (data not shown). We also found that after detachment by gentle pipetting, the 1205Lu MBs could reattach to a fresh surface. When re-plated in standard medium, MBs begin to collapse and produce a new monolayer (NM) resembling the original monolayer (OM) (Fig. 1D).

### Stem Cell-Like Transcriptional Signature in Cells Maintained as 3D-MBs

**Figure 2:**
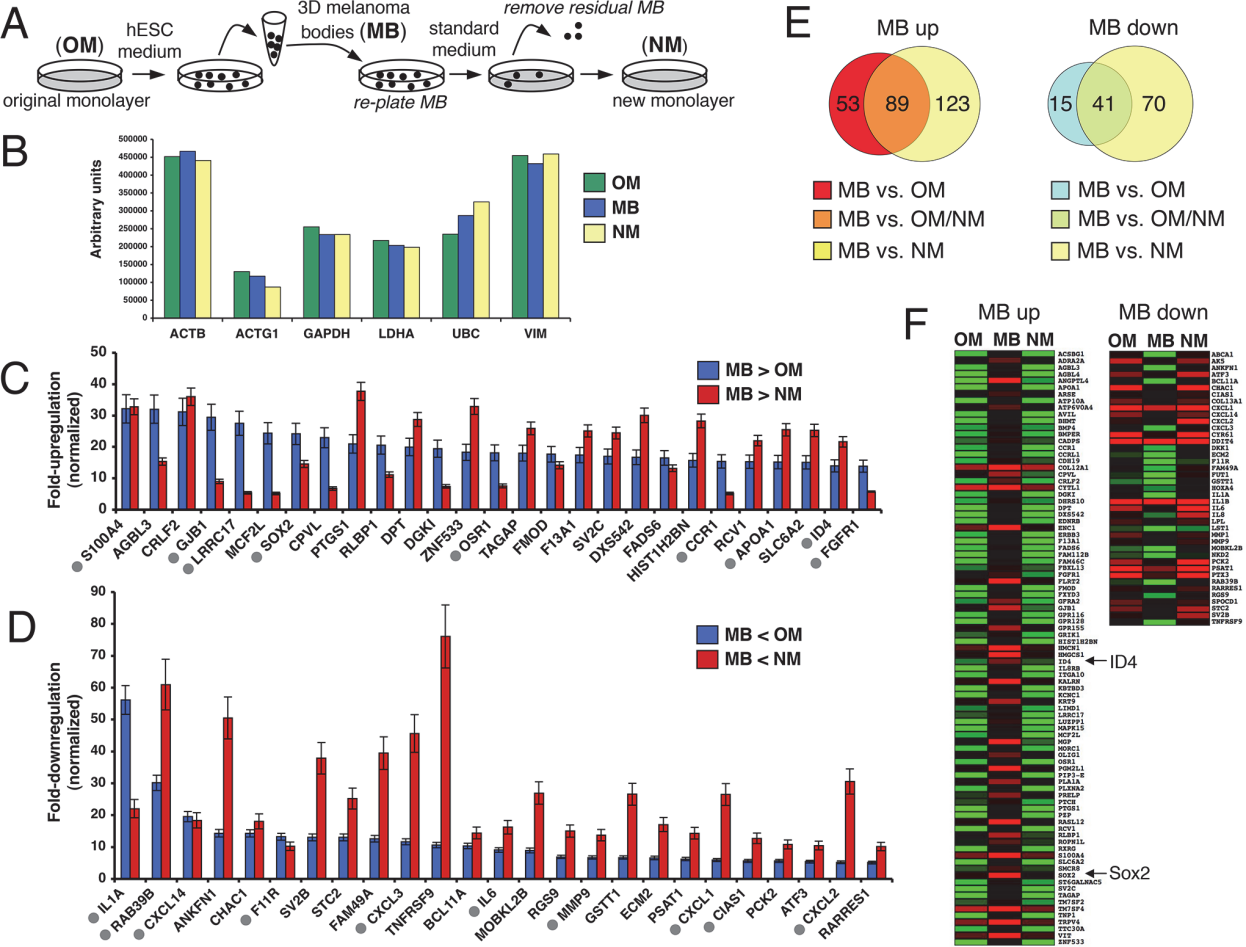
Expression microarray analysis of monolayer versus 3D-MB cultures. (**A)** Diagram of experimental design for comparison of transcriptomes of monolayer and 3D-MB cells. OM, original monolayer; MB, melanoma bodies; NM, new monolayer. **(B)** Raw microarray data for six housekeeping genes in OM, MB and NM RNA samples: ACTB, Beta-Actin; ACTG1, Gamma 1 Actin; GAPDH, Glyceraldehyde-3-Phosphate Dehydrogenase; LDH1, Lactate Dehydrogenase; UBC, Ubiquitin C; VIM, Vimentin. An average value was calculated from 10 spots, for each gene. (**C)** Sampling of genes upregulated 5-fold or greater in the MB sample compared to the OM and NM samples (MB>OM, MB>NM). Genes are ordered according to the fold-upregulation in the MB sample versus OM sample (MB>OM). Samples were normalized versus an average of three housekeeping genes, GAPDH, ACTB and HSP90 (see Panel B), and error bars represent the standard deviation. Grey filled circles indicate genes categorized with the GO term “developmental process.” (**D)** Sampling of genes downregulated 5-fold or greater in the MB sample compared to the OM and NM samples (MB<OM, MB<NM). Genes are ordered according to fold-downregulation in the MB sample versus OM sample (MB<OM). Grey filled circles indicate genes categorized with the GO terms “inflammatory response” or “cell motility.” (**E)** Venn diagram showing the number of upregulated and downregulated (unshared and shared) genes when OM, MB and NM are compared. (**F)** Heat maps of genes upregulated and downregulated in MB samples compared to OM and NM samples.

A large fraction of 1205Lu monolayer cells are capable of stem cell-like behavior as indicated by rapid formation of 3D-MBs through cell aggregation (Fig. 1A-C, S1 Fig.). This is a reversible process, as MBs can seed new monolayers (Fig. 1D). To identify factors that could potentially modulate this reversible, epigenetic-based phenotype-switching, gene expression microarray analysis was carried out on three purified cell populations (Fig. 2A): the original 1205Lu 2D-monolayer culture (OM), 14 day-old 3D-MBs collected by physical detachment (MB), and new 2D-monolayer (NM) cells produced from MBs after growth for 10 days in standard media, followed by removal of residual MBs.

The raw microarray data were robust and informative. First, six housekeeping genes were found to be expressed nearly equally among the OM, MB and NM samples (Fig. 2B). Second, small sets of genes were dramatically upregulated or downregulated in the MB sample as compared to the OM sample. For nomalization, gene expression levels were compared to an average of three house keeping genes (GAPDH, HSP90 and ACTB), with the criteria for differential expression being an average 5-fold change or greater between the MB and OM samples. Applying these criteria, 142 genes were upregulated and 56 genes were downregulated in MBs as compared to the OM sample (Table 1). A more stringent criteria was then applied by including 5-fold or greater upregulation or downregulation as compared to both the OM and NM samples (MB>OM plus MB>NM) (Fig. 2C-E). This reduced the number of genes that scored as upregulated in the MB sample from 142 to 89, and the number downregulated from 56 to 41 (Fig. 2E, S1 and S2 Tables). A heat map display shows the 89 genes upregulated and the 41 genes downregulated in MB the sample as compared to both the OM and NM samples (Fig. 2F).

**Table 1:** Initial criteria and summary for upregulated and downregulated genes detected by expression microarray.

	**MB vs OM**		**MB vs NM**	
	**MB up**	**MB down**	**MB up**	**MB down**
5-fold change	401 (0.9%)	424 (1%)	846 (1.9%)	614 (1.4%)
a.u. > 100	207 (0.4%)	135 (0.3%)	552 (1.2%)	215 (0.4%)
Known Genes	142 (0.3%)	56 (0.1%)	212 (0.4%)	111 (0.2%)

Comparisons were made relative to the melanoma body (MB) sample. OM, original monolayer; NM, new monolayer; a.u., arbitrary units. Percentages reflect the number of genes in each criteria as compared to the total number of genes analyzed.

**Figure 3:**
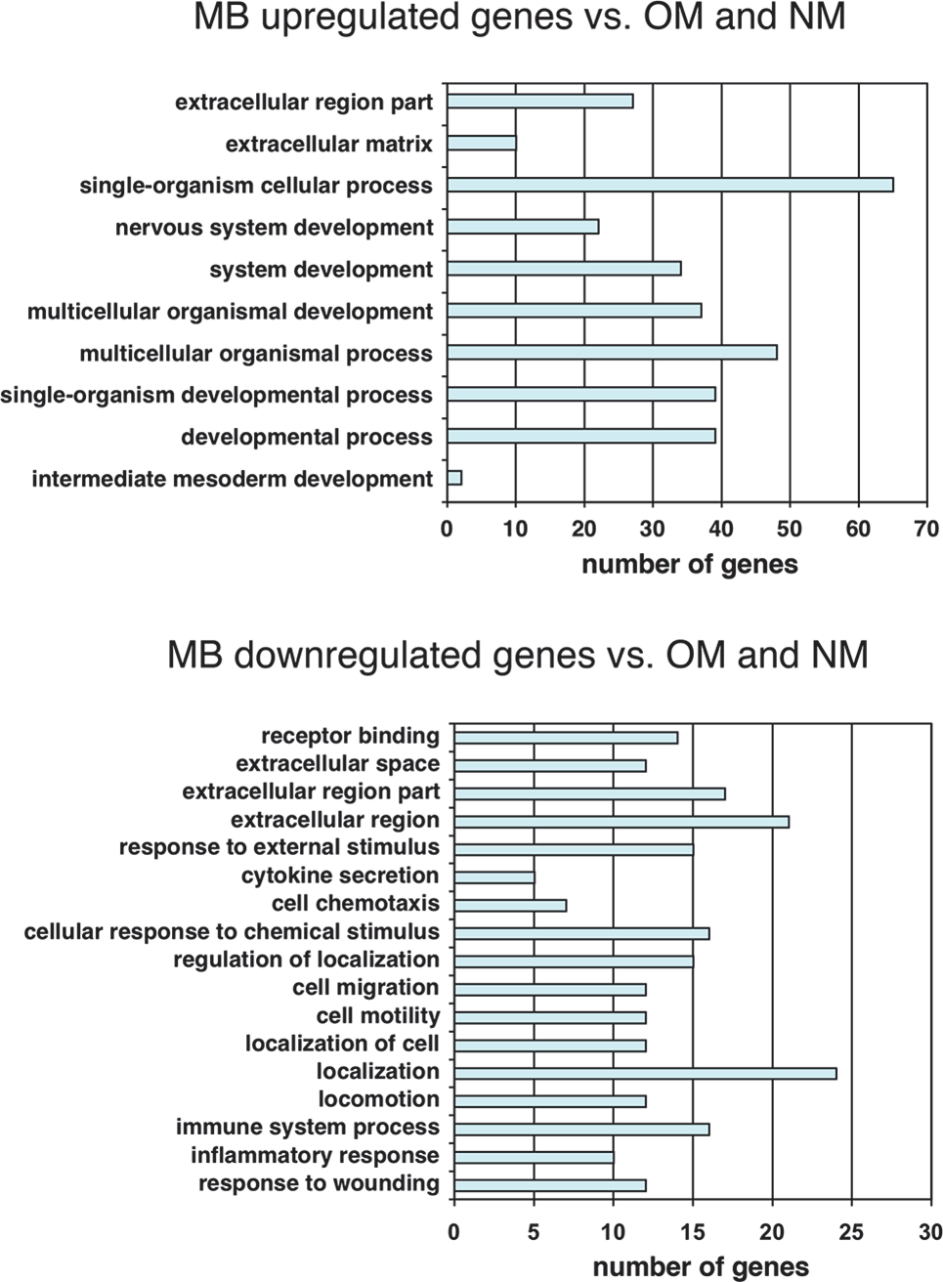
Gene Ontology analysis of genes upregulated and downregulated. GO analysis was performed to determine biological processes associated with MB-upregulated and MB-downregulated genes. Bars represent the number of regulated genes. MB-upregulated genes clustered mainly to developmental processes (top panel), while MB-downregulated genes clustered mainly to signaling, motility and invasion-related processes (bottom panel).

Strikingly, Gene Ontology (GO) analysis showed that 39 of the 89 MB-upregulated genes (S1 Table) were categorized as functioning in a “developmental process,” while 22 of the 41 MB-downregulated genes (S2 Table) were categorized as functioning in an “inflammatory response” or “cell motility.” Both gene sets included members of GO categories related to extracellular localization. Fig. 3 summarizes the GO analysis of the 89 MB-upregulated and 41 MB-downregulated genes. Fig. 2C and 2D are organized to highlight a sampling of the MB-upregulated and -downregulated genes based on scalability for display. The MB-upregulated set included neural stem cell genes; of particular interest were two genes, SRY-box 2 (SOX2) and Inhibitor of DNA Binding 4 (ID4), discussed in detail below. The MB-downregulated genes include cancer-associated cytokines/chemokines (e.g. CXCL1, CXCL2, CXCL3, CXCL14, IL1A, IL1B, IL6, IL8) and matrix metalloproteinases (MMP1,MMP9). Overall, MB-upregulated genes were related to developmental processes and stem cells, while downregulated genes were related to the invasive nature of cancer cells.

We were particularly interested in whether the expression levels of the 41 MB-downregulated genes were fully restored when the cells returned to monolayer growth (MB to NM transition). The fold-differences in expression of these 41 genes between the MB sample and the OM/NM samples were therefore compared. We found that the expression levels of nearly all of the 41 genes were restored in the NM samples, but more interestingly, the expression of 18 genes was increased in the NM over the OM sample (S2 Fig.). Of these genes, six were related to cellular invasiveness, motility, and inflammation (CXCL1, CXCL2, CXCL3, IL6, MMP9). These results suggest an exaggerated reacquisition of an aggressive cancer cell transcriptome as cells exit the MB state. The NM sample was harvested 10 days after return to standard growth medium, and it will be of interest to determine whether the observed differences in the levels of these transcripts in the NM versus OM samples are the result of transient versus stable epigenetic changes in gene expression. The expression of other genes also differed between the OM and NM samples (Fig. 2C-D), suggesting other epigenetic-based differences beyond those highlighted in S2 Fig.

The overall microarray results suggest that cellular microenvironment, in this case 3D-stem cell growth conditions, can promote phenotype-switching (e.g. reversible plasticity) of melanoma cells through dramatic changes in the transcriptome.

### Evidence For Functional Stem Cell-Like Behavior In MBs

**Figure 4:**
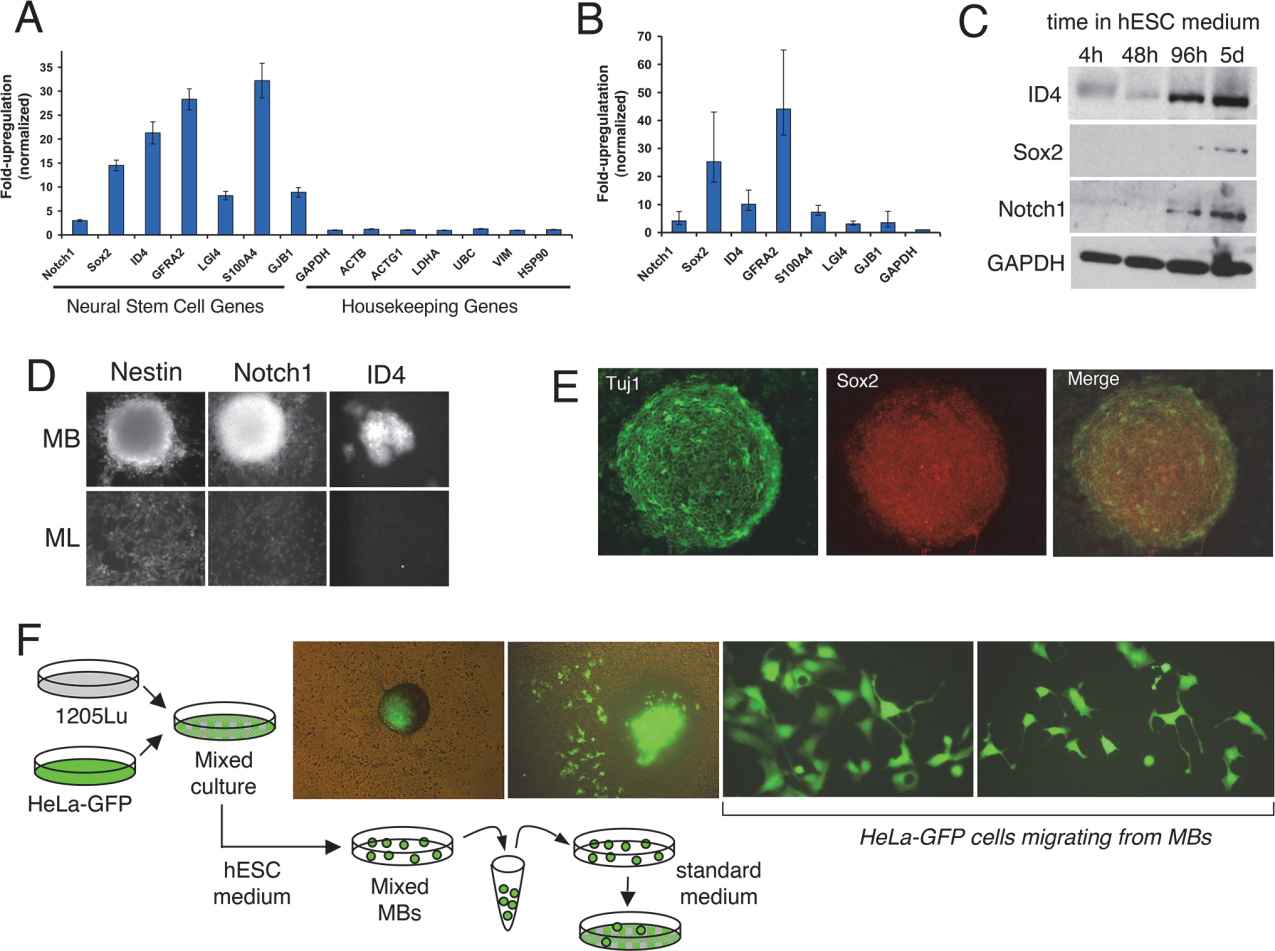
Neural stem cell features of 3D-MBs. (**A)** Data extracted from 1205Lu microarray analysis shows upregulation of neural stem cell genes in MB versus OM samples, normalized to average expression of three housekeeping genes, GAPDH, ACTB and HSP90. Error bars indicate standard deviation. (**B)** Analysis of neural stem cell gene upregulation in 1205Lu MBs using quantitative reverse transcription real time PCR, as normalized to GAPDH expression. Errors bars represent the standard deviation from triplicate samples. (**C)** Western blot analysis after switching 1205Lu monolayer cells to hESC conditions. (**D)** Immuofluorescence detection of neural stem cell markers in 1205Lu MBs and monolayer cultures. (**E)** Dual staining of MBs with TUJ1 and SOX2 antibodies. (**F)** Heterotypic MBs formed with 1205Lu and GFP-labeled HeLa cells were transferred to standard media, resulting in HeLa cells with neural features migrating from MBs.

The melanocyte, the cell type that gives rise to melanoma, is derived from neural crest cells, and neural gene expression is frequently observed in melanoma cells and cell lines [18,20,21,24,27,41,42]. The microarray results indicated that microenvironmental cues can trigger switching of melanoma cells to a neural stem cell-like pattern, as six genes related to neural stem cell and neural stem cell niche biology were upregulated: SOX2, ID4, GFRA2, S100A4, LGI4, and GJB1 (Fig. 4A). The neural stem cell gene NOTCH1 was upregulated ca. 8-fold when only the MB and NM samples were compared, but failed to meet the 5-fold criteria when the OM sample was included. Nonetheless, 5-fold or greater elevated expression levels of SOX2, ID4, GFRA2, S100A4, LGI4, GJB1, and NOTCH1 were confirmed by qRT-PCR analysis using OM and MB RNA samples (Fig. 4B).

We focused on the three MB-upregulated genes with roles in transcriptional control, NOTCH1, SOX2, and ID4, as they could potentially modulate phenotype-switching to the stem cell-like state. SOX2 is a transcription factor expressed in human embryonic stem cells and neural crest stem cells. ID4 is a member of the ID family of transcriptional repressors and is expressed in neural stem cells, while NOTCH1 controls transcription of a variety of cell fate genes. Western blotting showed that protein levels of NOTCH1, SOX2 and ID4 were elevated after shifting the 1205Lu monolayer cells to hESC medium for 96 hours, and these levels were maintained 6 days post-shift (Fig. 4C). 3D-MBs showed strong staining with antibodies against NOTCH and ID4, as well as NESTIN, a neural progenitor cell factor known to be variably expressed in 1205Lu cells and other melanoma cell lines [43–45] (Fig. 4D). Only weak staining for these proteins was observed in the corresponding original 2D-monolayer cultures (Fig. 4D).

3D-MBs also showed strong staining with antibodies against SOX2 (Fig. 4E). Dual staining of MBs was carried out with the TUJ1 antibody that recognizes an early neuronal-specific marker. The staining patterns indicated that a topological hierarchy exists within the 3D-MBs whereby cells located in the center express the stem cell marker SOX2, while cells at the periphery express the neuronal marker TUJ1 (Fig. 4E). Such organization is similar to that seen in normal 3D-stem cell growth, as well as in CSC spheroids [46–49]. These results suggest that 1205Lu cells can adopt stem cell-like functions within the 3D-MBs, whereby a central compartment of stem cell-like cells can give rise to a surface layer of more differentiated, neuronal-like cells.

Based on the findings above, 1205Lu MBs appear to represent functional structures, rather than cell aggregates, and are likely organized through cell-cell contacts, as well autocrine and paracrine signaling. An independent approach was employed to test this suggestion. Spheroids can assemble from mixed cell types, forming so-called heterotypic spheroids. HeLa cells do not form spheroids when grown in hESC medium (data not shown). However, co-culturing of GFP-marked HeLa cells with 1205Lu cells in hESC medium resulted in the assembly of heterotypic MBs containing both cell types (Fig. 4F). When these mixed MBs were shifted to standard media, HeLa cells that migrated from the MBs bodies acquired a dramatic neural-like phenotype. Previous studies have shown that HeLa cells can be transdifferentiated towards a neural phenotype, with neurite-like extensions and high migratory activity [50]. The experiments shown in Fig. 4E and 4F indicate that MBs, rather than behaving as simple cell aggregates, are functionally organized and have morphogenic inductive properties.

### ID4 Expression In Human Melanoma Tumor Samples

**Figure 5:**
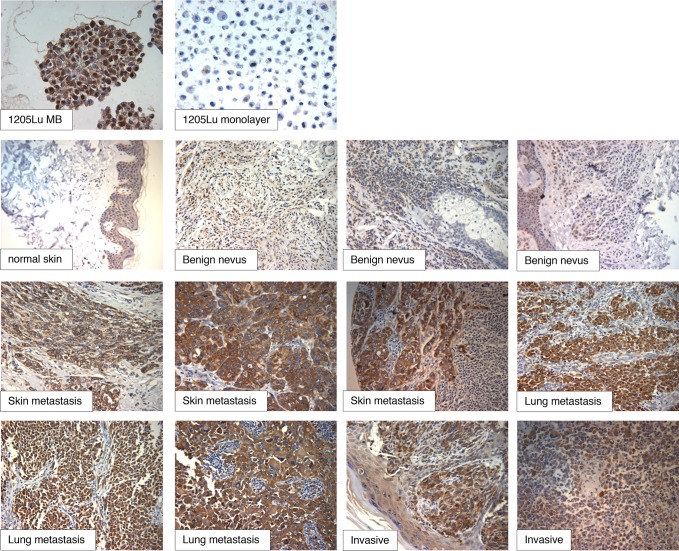
Staining of human melanoma tissue microarray samples with anti-ID4.

The next set of experiments was designed to test whether our experimental cell culture system could predict upregulated markers in human melanoma samples. Our focus turned to ID4, as SOX2 had been previously shown to be expressed in human melanoma tissues [13,20]. We first tested the effectiveness of the ID4 antibody in formalin-fixed, paraffin embedded samples using 1205Lu 3D-MBs and 2D-monolayer cultures. The results confirmed detectable levels of ID4 in monolayer cells, with a dramatic upregulation in the MBs (Fig. 5, top). The staining is consistent with western blot analysis comparing MBs and monolayers using the same antibody.

Next, human tissue microarray (TMA) slides were stained with the ID4 antibody. The slides contained 130 samples that included benign nevi, invasive melanoma, and metastatic melanoma. Intense ID4 reactivity was observed in nearly all melanoma samples, with only 8 out of 110 samples appearing negative (Fig. 5). Benign nevi showed much weaker and nonuniform ID4 staining as compared to the melanoma samples. Four out of five normal skin samples did not stain for ID4, while one sample showed weak staining in the epidermis (see Fig. 5). In this sample, the weak ID4 staining did not appear to correspond to any particular cell type within the epidermis. Magnified images of weakly staining, and non-staining, normal skin are provided in S4 Fig.

ID4 protein overexpression has been observed in triple negative breast cancer [51] and glioblastoma [52]. As opposed to the nuclear ID4 staining seen in these cancers, melanoma tissues showed largely cytoplasmic ID4 staining. Although the ID family of proteins are generally thought to function in the nucleus as repressive binding partners of bHLH nuclear transcription factors, they can shuttle to the cytoplasm where they might serve to block transport of bHLH proteins into the nucleus [53,54]. Similar to our findings, cytoplasmic ID4 staining has been observed in prostate cancer cells [55].

### SiRNA Knockdown of ID4 and SOX2 Block Formation of 3D-MB Structures

**Figure 6:**
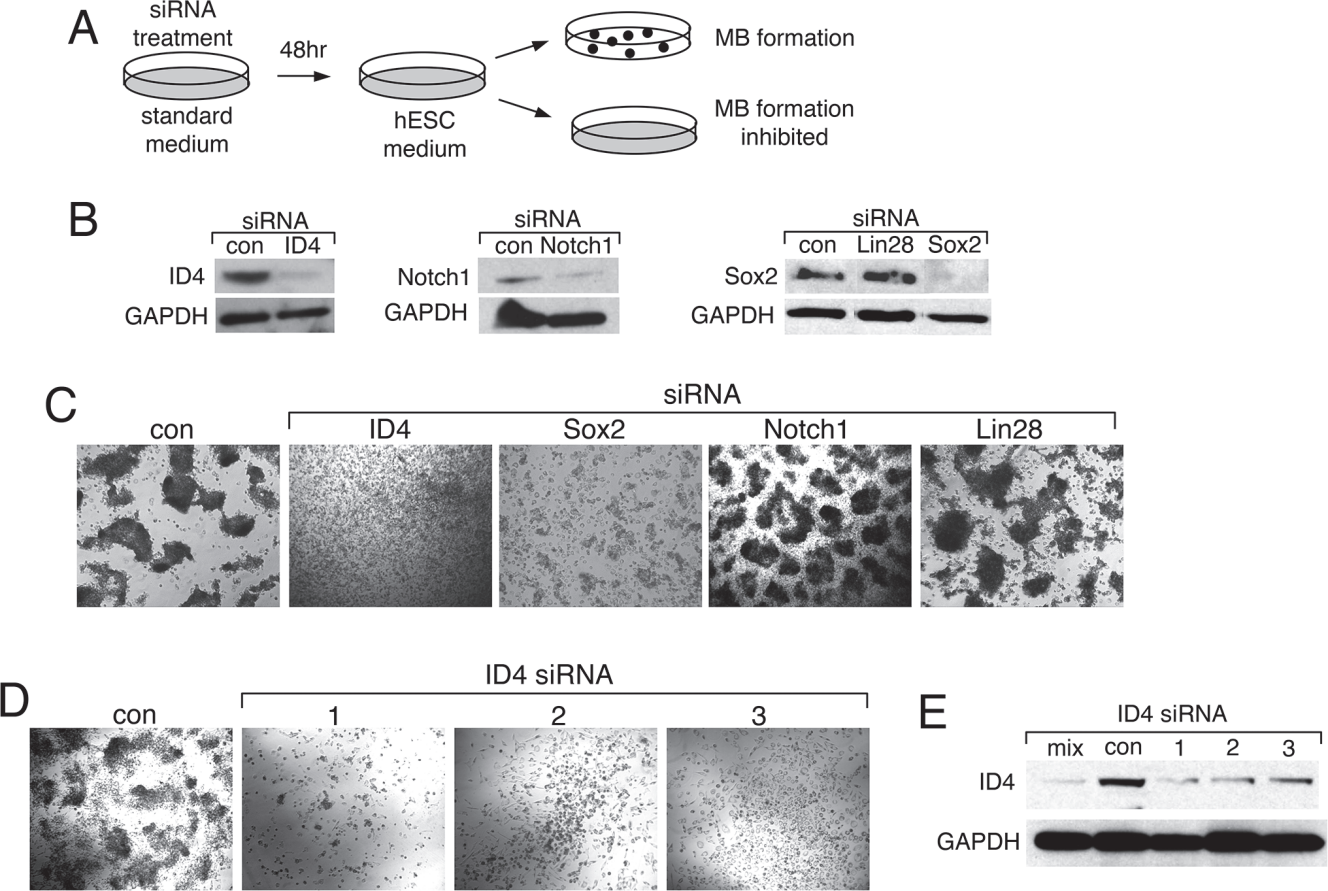
Knockdown of ID4 or SOX2 inhibit MB formation. **(A)** Diagram of experimental design. (**B)** Effectiveness of siRNAs in knockdown of ID4, SOX2 and NOTCH1 in hESC media. Western blots were carried out using LIN28 siRNA as a negative control. (**C)** The 1205Lu cells were treated with the indicated siRNAs, followed by challenge with hESC medium. MB formation was monitored. (**D)** The1205Lu cells were treated with individual ID4 siRNA, followed by challenge with hESC medium. Cell morphology was monitored after 7 days of culture in hESC media. (**E)** Western blots were carried out to analyze the effectiveness of individual ID4 siRNA. Lanes: mix, a mix of three siRNAs targeting ID4; con, untreated; 1,2,3, independent siRNAs targeting ID4.

The next set of experiments was designed to assess functional roles of NOTCH1, SOX2 and ID4 in the phenotype-switching demonstrated by 1205Lu cells, namely their ability to acquire stem cell-like features associated with 3D-MB formation. Specifically, we designed an siRNA-based knockdown experiment to determine whether upregulated MB-factors are required for MB formation (Fig. 6A).

The 1205Lu monolayer cells were treated with pools of four siRNAs targeting each of three candidate factors, NOTCH1, SOX2 and ID4. After 48 hours, the cultures were shifted to hESC media (with media replenishment every 24 hours), and were monitored over the next seven days for MB formation. Factor knockdown was confirmed by western blot analysis (Fig. 6B). SOX2 siRNA had an attenuating effect on mature MB formation, while NOTCH1 siRNA had no effect. However, knockdown of ID4 completely blocked the ability of cells to form MBs (Fig. 6C). Lin28 siRNA was chosen as a negative control, as our microarray analysis indicated that the Lin28 gene was not expressed at significant levels in either 1205Lu monolayer cells or in MBs. As expected, Lin28 siRNA knockdown had no effect on MB formation. Thus, ID4 knockdown, and to some extent SOX2 knockdown, specifically inhibited 3D-MB formation. The ID4 siRNA effect could be reproduced with three independent ID4 siRNAs, further demonstrating specificity (Fig. 6D and E).

**Figure 7:**
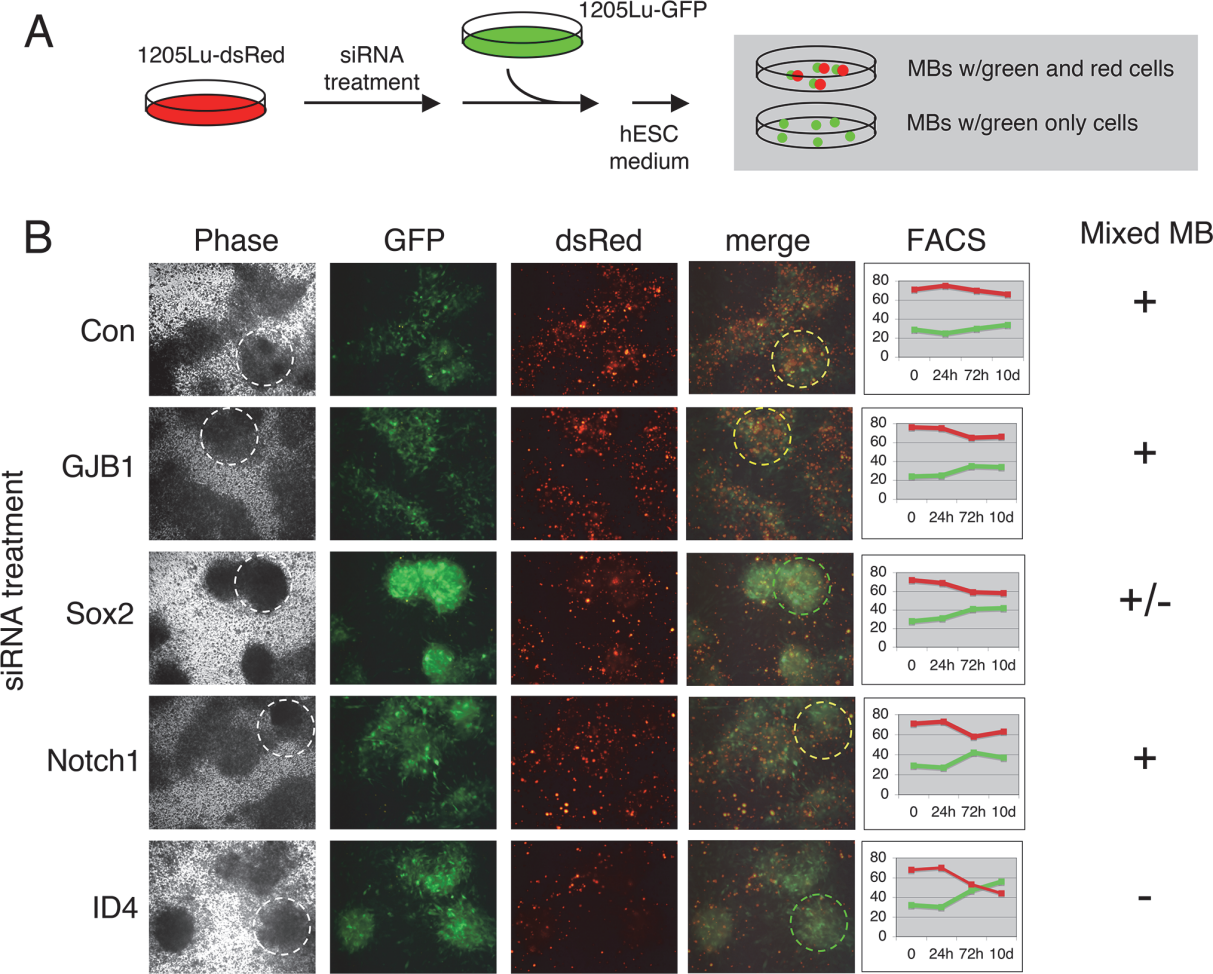
Analysis of defects in MB formation. **(A)** Diagram of experimental design. Nuclear DsRed-labeled 1205Lu cells were treated with siRNAs, mixed with untreated GFP-labeled 1205Lu cells, and plated under hESC conditions. (**B)** Imaging of MB formation. Merged image shows readout of red-green mixed (yellow dashed circle) versus green-only (green dashed circle) MBs. In the phase contrast image, the MBs corresponding to the merged images are indicated (white dashed circle). Right, FACS analysis was used to independently monitor cell growth or survival of green and red cells in hESC medium.

The mechanisms by which factor knockdown might inhibit 3D-MB formation included the loss of stem cell-like functions required to establish the MB structure, or simply effects on cell viability. We therefore asked whether knockdown cells that could not form 3D-MBs could still survive in hESC media and/or participate in the MB structures. We employed a cell-mixing strategy using the two 1205Lu cell populations that were differentially marked by dsRed-nuc (nuclear labeling) or whole cell GFP expression (Fig. 1C). In this way, the growth and behavior of siRNA knockdown cells could be monitored on a background of untreated cells that were capable of MB formation (Fig. 7A). For these experiments we focused on NOTCH1, ID4 and SOX2, and also included analysis of the MB-upregulated gap junction protein, GJB1.

The1205Lu-dsRednuc cells were treated with the indicated siRNA pools (Fig. 7B) for 48 hours. The cells were then switched to hESC media, and untreated 1205Lu-GFP cells were added. The hESC medium was replenished every 24 hours until MBs formed in the untransfected control cultures (Con) (Fig. 7B). MBs that formed from untransfected or NOTCH1 siRNA-treated 1205Lu-dsRednuc cells served as negative controls (see Fig. 6C), and showed red-green mixing in proportion to their respective input percentage (ca. 65% red and 35% green) (Fig. 7B). Consistent with the results in Fig. 6C, SOX2 siRNA knockdown resulted in a diminished red cell fraction in MBs, while ID4 siRNA treatment resulted in a dramatic absence of red cells in MBs (Fig. 7B) indicating that ID4-knockdown cells could not participate with ID4-expressing cells (green control cells) in MB formation. Lastly, although GJB1 siRNA knockdown produced a defect in MB formation (data not shown), GJB1-knockdown cells could participate in MB formation (Fig. 7B).

The percentages of siRNA-treated (dsRednuc) and untreated (GFP) cells were monitored over time by FACS during this experiment to identify potential defects in proliferation or survival of ID4 and SOX2 knockdown cells that could account for their reduced presence in MBs (Fig. 7B). As shown, a reduction in red cell numbers was not sufficient to account for the partial or complete absence of red cells in MBs after SOX2 or ID4 knockdown. However, ID4 siRNA treatment appeared to have the most impact, with 30% fewer red cells present in the culture at 10 days as compared to the control.

### ID4 Knockdown Results in Progression of 1205Lu Melanoma Cells To a More Differentiated Phenotype

In several experiments in which 1205Lu ID4-knockdown cells were transferred to hESC medium (see Fig. 6A), we observed the dramatic appearance of cell differentiation features. That is, rather than hESC medium triggering the stem cell-like MB state, ID4-deficient cells appeared to proceed to a more differentiated state. Furthermore, we suspected that the reduced number of the red ID4 siRNA treated-cells in the mixing experiments (Fig. 7B) might reflect the reduced proliferation that accompanies cell differentiation.

**Figure 8:**
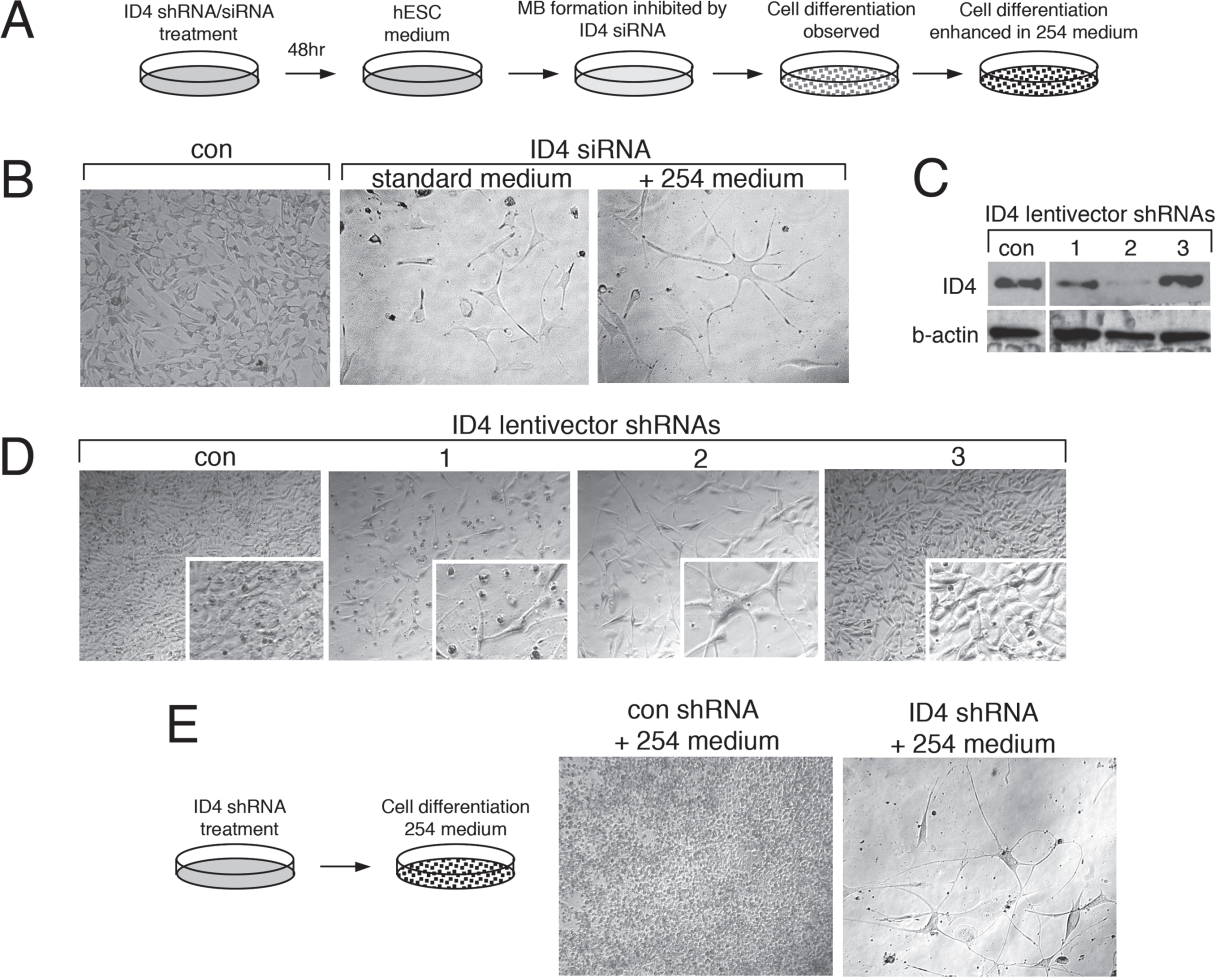
Knockdown of ID4 promotes differentiation of 1205Lu cells. **(A)** Diagram of experimental design. (**B)** Representative images showing differentiation phenotype of 1205Lu cells after ID4 siRNA knockdown, hESC media challenge (five days), and switching to 254 medium (96 hours). (**C)** Western blot testing of ID4 knockdown using shRNAs delivered by lentivectors. **(D)** Representative images showing differentiation phenotype of 1205Lu cells after ID4 shRNA knockdown and hESC media challenge. (**E)** Representative images showing differentiation phenotype after ID4 shRNA delivery to 1205Lu cultures without exposure to hESC medium. Phenotype was enhanced after 96 hours in 254 medium.

Cells that failed to form MBs after ID4-knockdown appeared more spindle-shaped, and some showed long neural-like processes (Fig. 8A and B). We tested media conditions that might stabilize or enhance this differentiated phenotype. As such, once this spindle-shape morphology was observed, cells were transferred to either standard 1205Lu growth medium or specialized media. Standard media appeared to stabilize the spindle-shaped ID4-knockdown cells, but a more striking differentiation phenotype was observed in 254 melanocyte medium (Fig. 8B), as cells demonstrated long processes and higher levels of dendricity.

**Figure 9:**
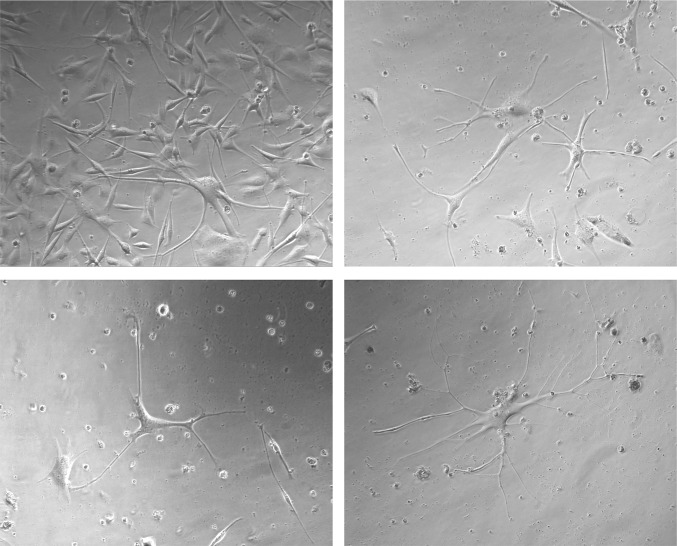
Representative images showing differentiation phenotype of 1205Lu cells after ID4 shRNA delivery, followed by growth in 254 medium for 96 hours.

Although ID4 was highly upregulated in MBs, there was also detectable expression in 1205Lu monolayer cells (Fig. 4D and data not shown). We therefore next used lentivectors expressing ID4 shRNAs in an attempt to construct stable 1205Lu ID4-knockdown cells. The 1205Lu cell cultures were infected with lentiviral vectors encoding three independent shRNAs targeting ID4, as well as a control lentivector encoding a nonspecific shRNA sequence. As shown in Fig. 8C, the shRNAs showed variable effectiveness in knocking down ID4 (shRNA #2 > shRNA #1 > shRNA #3). We found that cells selected for stable ID4 knockdown with shRNA #2 rapidly demonstrated a more differentiated phenotype (Fig. 8D). Consistent with the knockdown efficiencies shown in Fig. 8C, ID4 shRNA#1 produced a mild differentiated phenotype, while shRNA#3 shRNA had no effect. The stable 1205Lu ID4 shRNA#2 knockdown cells also showed increased CFSE dye-retention as compared to controls, consistent with the slower growth rate expected for moving towards to a more differentiated state (S3 Fig.). Finally, switching of shRNA#2 knockdown cells to 254 medium again resulted in a dramatic phenotype as compared to cells treated with control shRNA (Figs. 8E and 9). Thus, ID4 appears to be key in restricting the transition of 1205Lu to a more normal, differentiated state.

## Discussion

Although numerous insights into melanoma biology have been made in the past thirty years, the exploration of new model experimental systems is needed to address emerging disease features and accompanying hypotheses, in particular with respect to the origins of heterogeneity. Although some recent work, described in the Introduction, has not supported a CSC model for melanoma, melanoma cells can express stem cell markers and display stem cell properties. We agree that melanoma cells that acquire stem cell features are best described as “stem-like” cells, indicating that they share some properties with normal stem cells, while not implying that they are CSCs [22]. Rather than playing roles as true CSCs, our observations suggest that stem-like cells in melanoma can serve as intermediates. The reversible phenotype-switching that we have observed has revealed cellular capabilities that might contribute to tumor heterogeneity and adaptability. Indeed melanoma cells in culture are able to switch phenotypes after clonogenic purification, resulting in mixtures of cell types, including stem cell-like and differentiated phenotypes [45]. Studies with other cancer models have indicated that incompletely differentiated cancer cells can dedifferentiate and acquire stem cell-like features [22,23,56]. Microenvironmental cues can also trigger stem/progenitor cell programming in melanoma, thereby hijacking developmental pathways to promote cancer hallmarks such as cell migration and invasion [10,21,24]. The re-expression of the embryonic Nodal signaling pathway is another example of exploitation of developmental pathways in melanoma [7]. Our intent was to develop a cell culture system that would functionally synchronize cultured melanoma cells in the most primitive stem cell-like state possible, and then identify genes that might regulate such phenotypic switching. We believe that if stem cell-like melanoma cells are important plastic intermediates, the genes that control phenotype-switching might serve as novel functional targets for therapy.

Primary melanoma cells, or cell lines, can be propagated as floating 3D spheroids [35]. Melanoma spheroid formation is typically promoted by simply blocking cell attachment, but floating spheroids will also form under stem cell conditions [28]. Numerous findings have indicated that melanoma spheroid cells have specialized properties, distinct from the monolayer from which they were derived. These properties include the ability to differentiate into a number of cell lineages when shifted to specialized growth media [19,24,27,35,36,57,58]. In melanoma cell culture systems, as well as numerous other cancers, 3D spheroid growth has also been reported to enrich for CSCs or TICs [35]. However, the usefulness of spheroids as an indicator of melanoma CSC behavior has been questioned [36]. Distinct from floating melanoma cell spheroids, we found that 1205Lu monolayer cells could form adherent spheroids (MBs) in hESC medium. This ability provided a technical advantage to study melanoma cell phenotype-switching, as cultures could be transitioned from 2D-monolayer growth, to 3D-stem cell-like spheroid growth, and then back to 2D-monolayer growth. Our goal was to monitor melanoma cells as they transit on this axis of distinct levels of differentiation, and thereby gain knowledge of factors that regulate these cellular dynamics.

Comparing the transcriptomes of 1205Lu cells grown as 3D-MBs versus 2D-monolayers revealed that only a small fraction of genes were differentially expressed based on our criteria. These differentially expressed genes in MBs (both upregulated and downregulated) were functionally clustered in a manner consistent with the predicted stem-like state of MB cells (Figs. 2 and 3). The MBs showed upregulation of developmental genes, and this profile shared features with both normal neural stem cells and glioma cancer stem cells [59]. Thus, our results join others that showed upregulation of developmental genes in melanoma spheroids [24].

Interestingly, MB cells showed downregulation of genes related to the invasive phenotype of melanoma (e.g., MMPs, cytokines, chemokines). This finding is seemingly at odds with previous reports that have demonstrated that cells from melanoma spheroids are more invasive and tumorogenic than their monolayer counterparts [27]. Of particular note, spheroid-derived 1205Lu cells have been previously demonstrated to be highly invasive [60]. However, we observed that after shifting 3D-MBs cells out of hESC medium to allow formation of a new 2D-monolayer (the NM sample), there was a coordinated reacquisition of invasive markers. Furthermore, our expression microarray data indicated enhanced upregulation of genes associated with invasiveness in the NM sample (S2 Fig.). We suggest that the tumorigenicity and invasiveness observed with spheroid-derived melanoma cells could be attributable to the rapid reacquisition of a highly aggressive phenotype as the cells exit the stem cell-like state. This may indicate a strategy for melanoma tumors to expand and seed metastasis using a stem-like cell intermediate, whereby cells might reacquire a “super aggressive” phenotype when environmental signals trigger exit from the stem-like state. In contrast to our findings, an earlier study [61] did not detect a stem cell transcriptome signature in melanoma spheroid cells, but rather an invasiveness signature. However, these spheroids were formed by blocking cell attachment rather than through hESC conditions. Indeed, a more recent report demonstrated differences in melanoma spheroids that are formed under stem cell conditions versus those formed by preventing attachment [37].

The neural transcriptional signature in stem-like 1205Lu MB cells (NOTCH1, SOX2, ID4, GFRA2, LGI4, S100A4, GJB1) might be expected, given that melanocytes originate from neural crest cells. Among the upregulated genes was S100A4, a member of the S100 protein family of proteins that serve as markers for human melanoma [5]. As mentioned, SOX2 has also been detected in human melanoma [6,13,19,20]. Our results also indicate that the transcriptional profile of MBs may signify the presence of functional stem-like cells as demonstrated by their plasticity and morphogenic activity. As shown in Fig. 4, and discussed above, staining of MBs with SOX2 and TUJ1 antibodies revealed a functional organization of cells within the MB. Furthermore, the 1205Lu MB environment could promote a striking neuronal morphology in HeLa cells (Fig. 4F), similar to that observed after overexpression of the neural developmental regulator Pax6 [50].

Based on the findings above, we considered that the MB-specific transcriptome might provide a means to identify stem cell factors upregulated in human melanoma. Indeed, a major finding of our work is that ID4 is highly expressed in a large fraction of melanoma tissues (Fig. 5). As is the case with other common melanoma markers [5], ID4 was also detectable in benign nevi, albeit at lower levels.

The ID4 family of transcriptional regulators share a leucine zipper domain with the bHLH transcription factors, but lack a DNA binding domain; these regulators inhibit bHLH transcription factors through formation of heterodimers that cannot bind to DNA [62,63]. ID4 has been previously associated with other human cancers, as both an oncogene and tumor suppressor gene [51,52,64–74]. One recent study has detected ID4 as an oncogene in ovarian cancer, and demonstrated that it is a valid therapeutic target [73]. This ovarian cancer study, and others, have provided some insights into the possible mechanistic roles of ID4 in cancer [67–69,75]. In our work, we detected ID4 as part of the upregulated stem cell-like expression profile in 3D-MB-derived cells. We used siRNA knockdown to investigate whether ID4 and other upregulated factors had potential “driver” functions in the observed reversible phenotypic switching. Transient ID4 siRNA knockdown completely blocked MB formation at an early step prior to cell aggregation, and knockdown cells were incapable of participating in MB formation with ID4-expressing cells (Fig. 6C and D, Fig. 7B). In contrast, immature MB cell aggregates were formed after SOX2 siRNA knockdown and these knockdown cells could still participate in MBs to some degree (Fig. 6C, Fig. 7B).

After transient ID4 siRNA knockdown, a striking differentiated neural-like phenotype was observed (Fig. 8), and this likely contributes to the inability of ID4-depleted cells to participate in MB formation (Fig. 7B). Although the ID4 knockdown cells showed this neural-like phenotype, we were unable to detect expression of any specific neuronal markers, such as TUJ1 (data not shown). However, a reduction in cell number after ID4 knockdown, suggests that slower proliferation might accompany the differentiation phenotype (Fig. 7B, S3 Fig.). Relevant to the neural-like phenotype enabled by ID4 knockdown, ID4 is known to regulate neuro-ectodermal development, neural differentiation, as well as adult neural stem cell processes [76,77]. In these settings, and in concordance with our results, ID proteins have been described as “inhibitors of differentiation” [76,78,79], and their function is to maintain an undifferentiated cell state until the appropriate time in development is reached.

**Figure 10:**
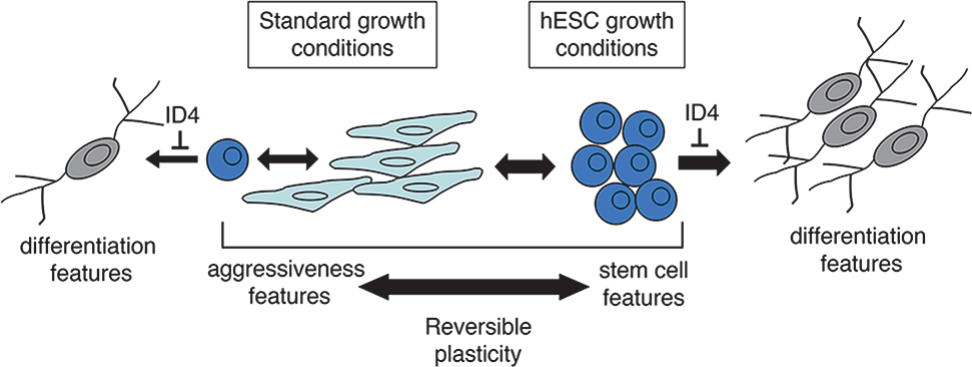
Model for role of ID4 as a differentiation guardian. Model proposes that melanoma cells can undergo phenotype-switching to acquire stem-like cell features. Growth in hESC media can synchronize this behavior. The proposed role of ID4 as part of the stem cell-like transcriptional signature is to act as a “differentiation guardian” to prevent progression of cancer cells to a more normal differentiated state.

We found that stable ID4 shRNA knockdown in 1205Lu cultures grown in standard conditions resulted in a uniform progression towards a normal differentiated state, and this effect could be enhanced using differentiation medium (Fig. 9). Similar, although less dramatic phenotypic changes were observed in other melanoma cell lines after ID4 shRNA treatment (data not shown). We describe the implication of this study’s findings in Fig. 10, as follows. ID4 is expressed at low levels in monolayer 1205Lu cells grown under standard conditions (Figs. 4 and 5). This low ID4 signal may signify a subset of cells that have spontaneously acquired stem cell-like transcription signatures and features. Under these standard growth conditions, shRNA knockdown of ID4 might interrupt phenotype-switching from the proliferative monolayer to stem-like cell states, and promote cell transit towards a default, more differentiated neuronal state. ID4 stable shRNA knockdown might thereby “trap” cells in the more differentiated state, and such cells would be expected to accumulate over time. That is, as ID4-deficient cells spontaneously shift to a more stem-like state, they would lose stem cell features and differentiate. Shifting the 1205Lu cultures to hESC medium appears to “synchronize” cells in this stem-like state, and thereby overall ID4 expression is dramatically increased as part of the stem cell signature (Fig. 4C).

In human melanomas, it appears that an embryonic pathway of differentiation produces areas of neuronal-like cells that participate in angiogenesis [80]. These findings suggest that neural differentiation of melanoma cells can indeed occur in specific areas within tumors. To our knowledge, there are no data addressing how the presence of such cells relates to aggressiveness of disease or patient survival. We suggest that therapeutic strategies aimed at driving widespread differentiation within the tumor may be useful. Our findings indicate that ID4 may be one of many targets, whose inhibition can result in progression to a more differentiated, neural-like state. The neural-like differentiated cells observed after ID4-knockdown were rapidly lost, even after culturing in differentiation medium. As such, we were unable to perform assays to test the prediction that these cells are less invasive then the parental monolayer cells. Future studies will be aimed at deriving culture conditions for these cells, identifying differentiation markers, and investigating their epigenetic stability.

We hypothesize that the developmental function of the ID4 gene as an inhibitor of differentiation is being exploited as a restrictive factor by melanoma cells to inhibit widespread terminal differentiation. The fraction of human melanoma tumor cells expressing ID4 was very high (Fig. 5), and this is generally consistent with our tissue culture model, where a majority of cells can acquire high ID4 expression under specific conditions (Fig. 4D). We suggest that the melanoma cell stem-like state provides several advantages, possibly in dissemination, or contributing to a highly plastic, adaptable state. Indeed, a critical feature of melanoma may be microenvironmental triggering of such an advantageous stem-like state. In this model, ID4 would serve as a “differentiation guardian,” functioning in a dynamic “as-needed” role as part of the stem cell signature to block transit of stem-like cells to a more differentiated normal state. In our model, ID4 would thereby act as an oncogene. A very recent study detected low levels of ID4 in 1205Lu cells and provided evidence that ID4 is indeed an oncogene [81]. These observations are generally consistent with our study, but further work will be required to link the findings.

In summary, in this work we used a melanoma cell line-based system to study processes that occur as melanoma cells move on the axis between stem cell-like and proliferative states, and identified ID4 as having a functional role in phenotype-switching. Disabling of ID4 leads to differentiation of human melanoma cells. Differentiation therapy is long standing concept, developed in the 1970s, which has been found to be quite promising in hematological cancers [82]. This approach exploits the principle that cancer cells are blocked in an incompletely differentiated state. The system developed in this study has the potential to reveal previously unidentified factors that may serve as targets or functional markers in melanoma diagnostics, prognostics, or therapy. Our current study suggests that oncogenic ID4 may very well constitute a putatively novel melanoma target. Efforts are underway to initiate broad siRNA-based screening to identify additional modulators of melanoma cell phenotype-switching.

## Supporting Information

S1 Fig Growth of 1205Lu cells in standard medium versus hESC medium. **A** Cells were grown in standard or hESC media for 24 hours, after which time they were pulse labeled with Carboxyfluorescein Succinimidyl Ester (CFSE) dye. Dye retention was measured by flow cytometry after four hours or five days in each medium. **B**. Cell cycle assays based on DNA content were carried out after 24 and 96 hours in hESC medium or standard medium.
(TIF)

S2 Fig Increased expression of a gene set associated with the NM sample versus the OM sample. Results are extracted from the expression microarray data, and are shown as fold-upregulation in the OM and NM samples as compared to the MB sample. Filled red circles indicate genes associated with aggressive features of cancer cells.
(TIF)

S3 Fig Effect of ID4 shRNA knockdown on cell growth. The 1205Lu cells were grown in standard media and infected with lentivirus vectors encoding ID4 or control shRNAs. Cell growth was monitored by dye retention as described in S1 Fig. Cells were pulsed with CFSE for one hour and FACS analysis was carried out after four hours or five days.
(TIF)

S4 Fig Staining of normal skin with anti-ID4. Left panel show digital magnification of the normal skin sample shown in Fig. 5. Right panel shows a second sample of normal skin that is negative for ID4 staining.
(TIF)

S1 Table MB-upregulated genes from the expression microarray. A total of 89 genes were upregulated. The fold-upregulation versus both the OM and NM samples is provided. The criteria used for inclusion in this list was that expression was at least 5-fold greater in MB samples than both the OM and NM samples (MB>OM, MB>NM).
(DOC)

S2 Table MB-downregulated genes from the expression microarray. A total of 41 genes were downregulated. The fold-downregulation versus both the OM and NM samples is provided. The criteria used for inclusion in this list was that expression was at least 5-fold lower in MB samples than both the OM and NM samples (MB<OM, MB<NM).
(DOC)

S1 Methods Materials And Methods for experiments shown in S1 and S3 Figs. (DOC)
