# Supplementary figures and images for: Inhibitor of DNA Binding 4 (ID4) Is Highly Expressed in Human Melanoma Tissues and May Function to Restrict Normal Differentiation of Melanoma Cells
Source: PLoS One. 2015 Feb 2;10(2):e0116839. doi: 10.1371/journal.pone.0116839 (PMC4314081; doi:10.1371/journal.pone.0116839)

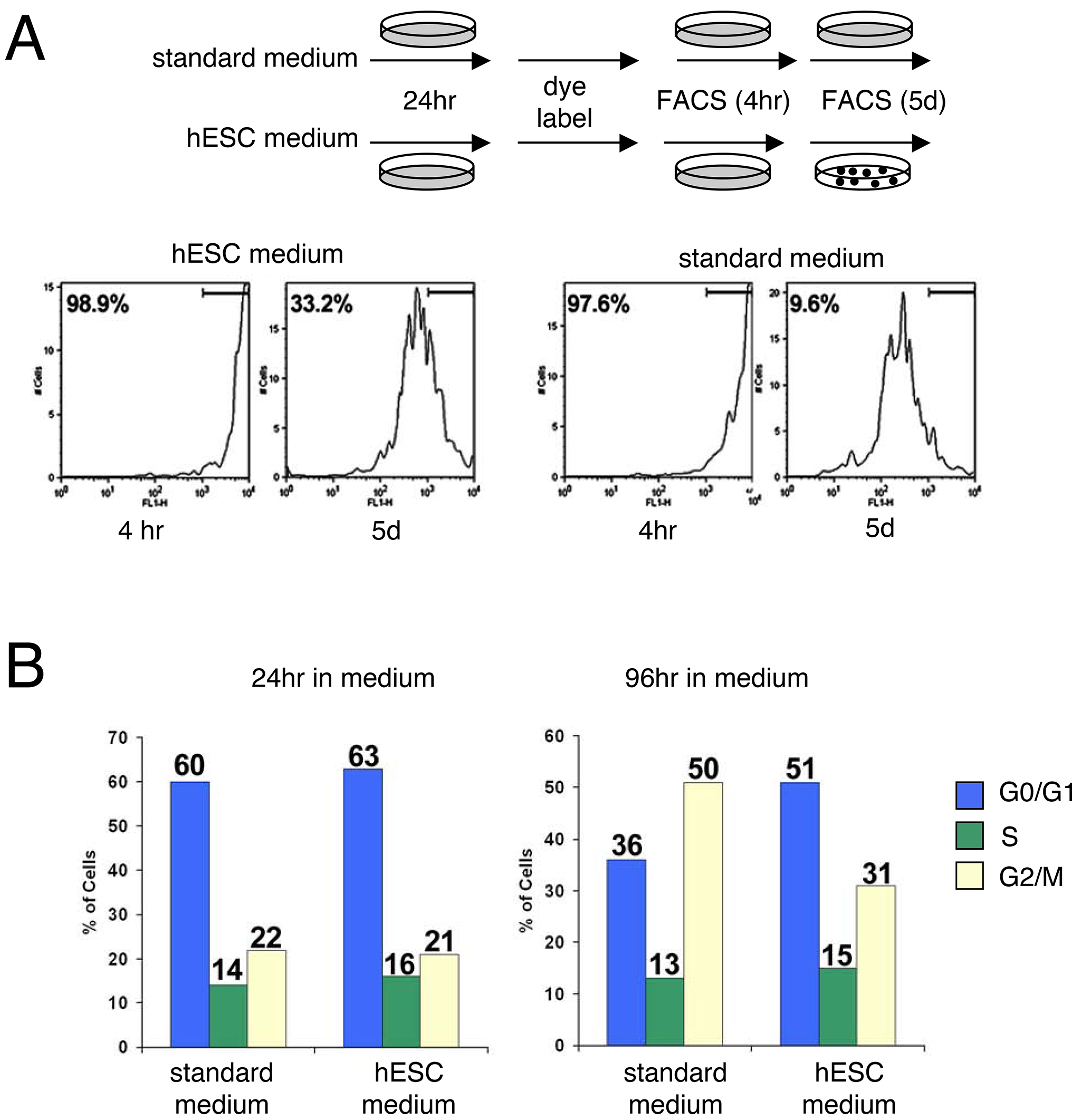

Supplement: S1 Fig — A Cells were grown in standard or hESC media for 24 hours, after which time they were pulse labeled with Carboxyfluorescein Succinimidyl Ester (CFSE) dye. Dye retention was measured by flow cytometry after four hours or five days in each medium. B. Cell cycle assays based on DNA content were carried out after 24 and 96 hours in hESC medium or standard medium. (TIF) [file pone.0116839.s001.tif]

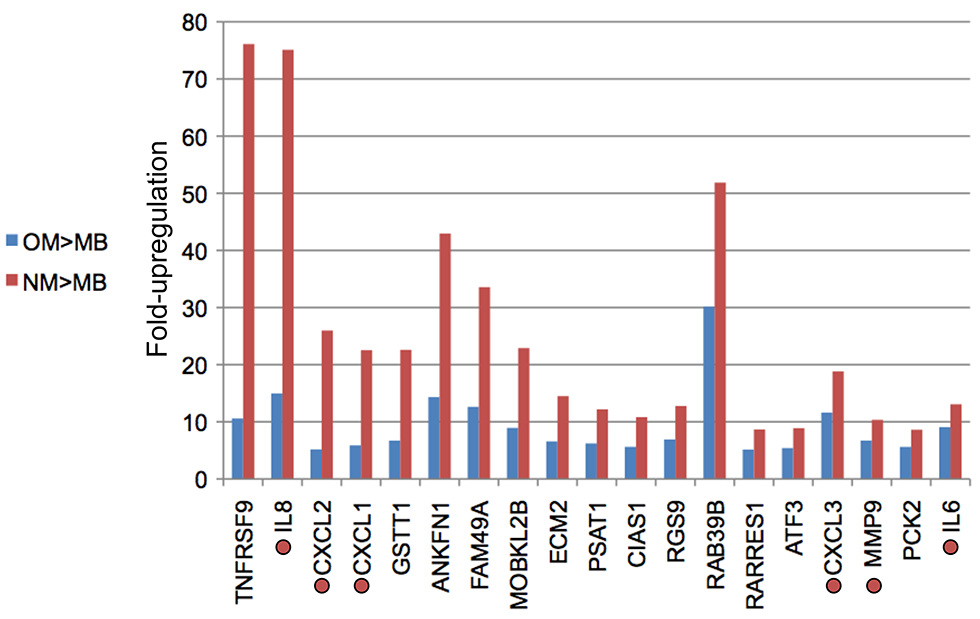

Supplement: S2 Fig — Results are extracted from the expression microarray data, and are shown as fold-upregulation in the OM and NM samples as compared to the MB sample. Filled red circles indicate genes associated with aggressive features of cancer cells. (TIF) [file pone.0116839.s002.tif]

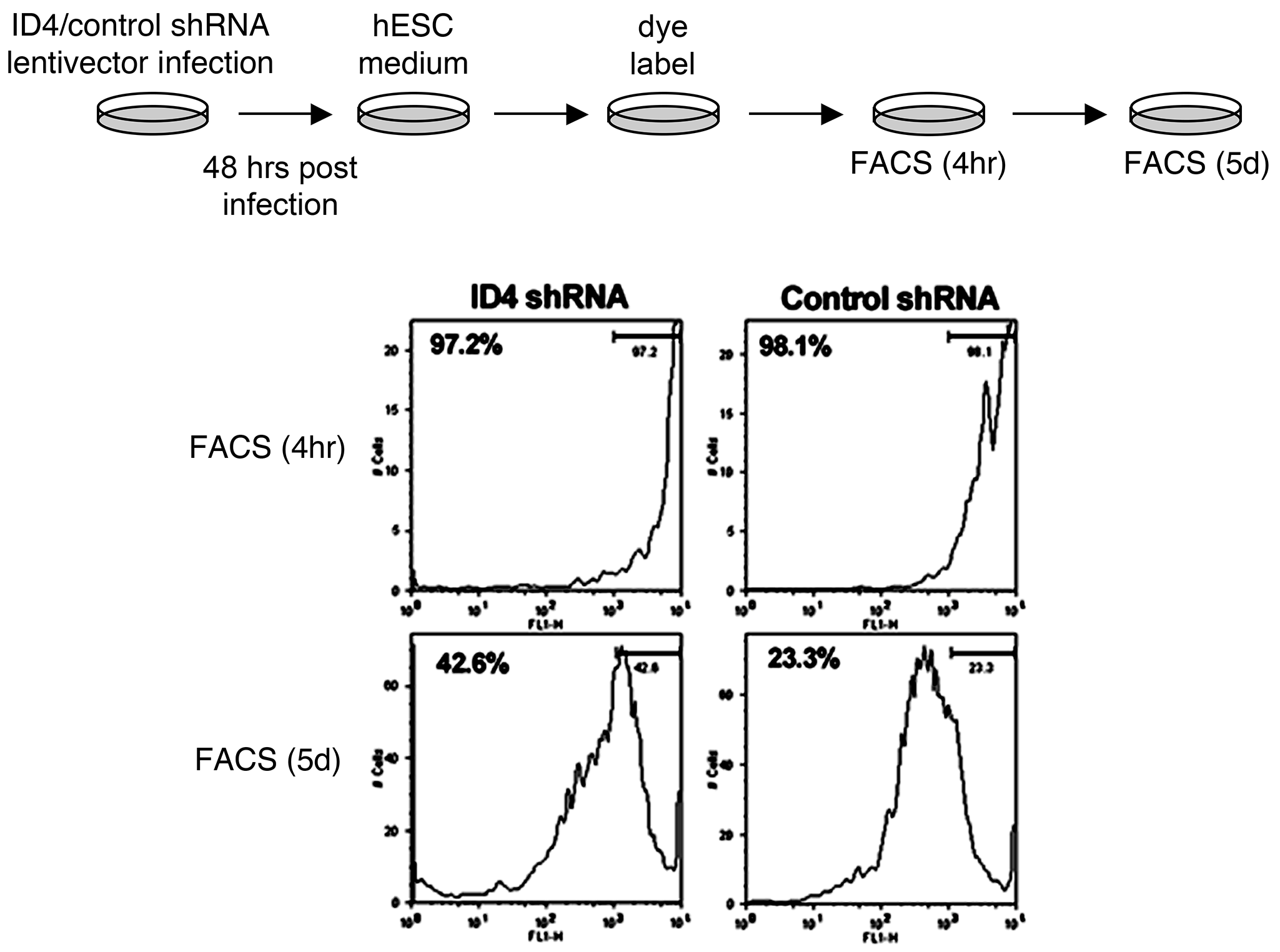

Supplement: S3 Fig — The 1205Lu cells were grown in standard media and infected with lentivirus vectors encoding ID4 or control shRNAs. Cell growth was monitored by dye retention as described in S1 Fig. Cells were pulsed with CFSE for one hour and FACS analysis was carried out after four hours or five days. (TIF) [file pone.0116839.s003.tif]

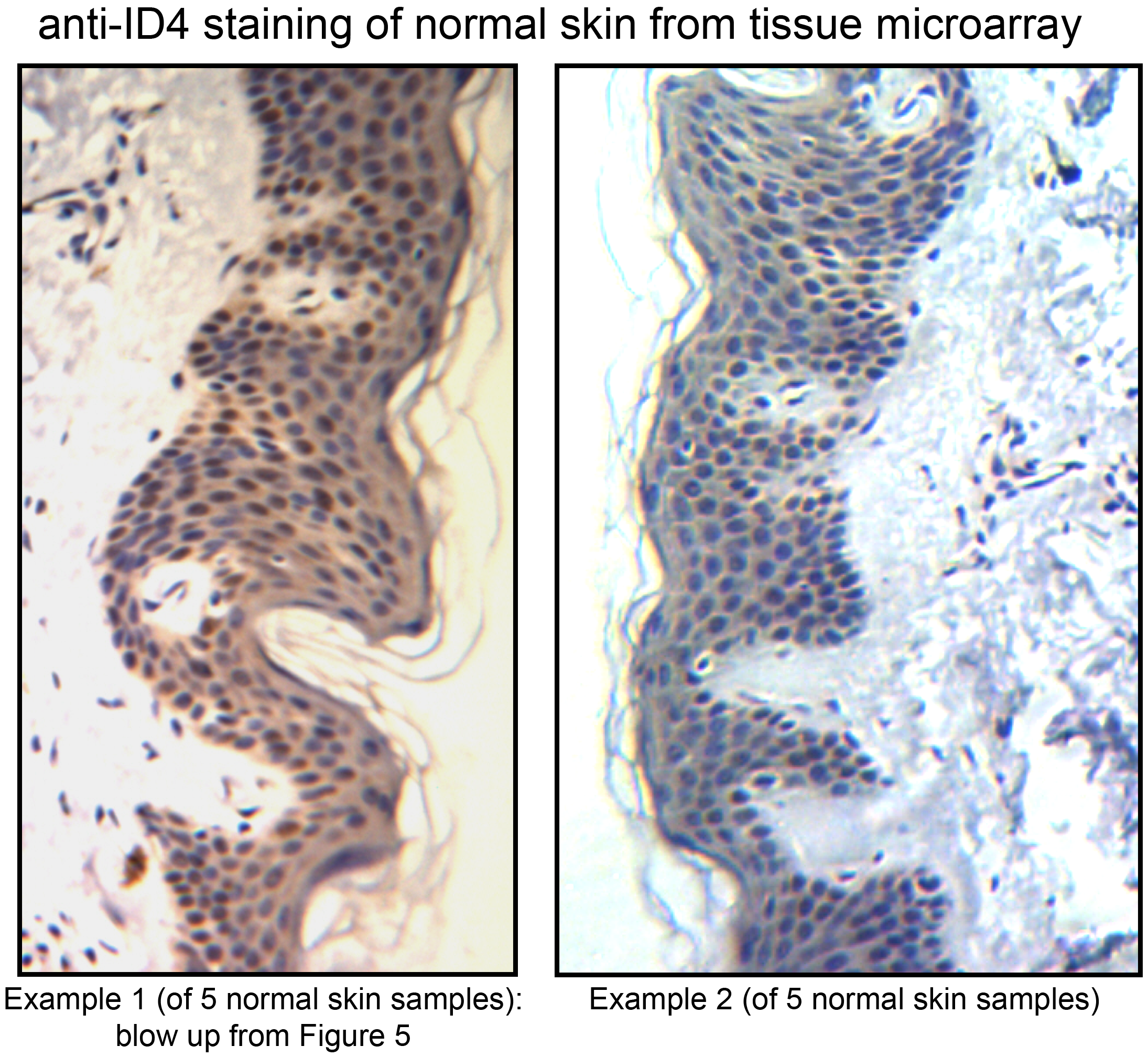

Supplement: S4 Fig — Left panel show digital magnification of the normal skin sample shown in Fig. 5. Right panel shows a second sample of normal skin that is negative for ID4 staining. (TIF) [file pone.0116839.s004.tif]
