# Supplementary material for: Inhibitor of DNA Binding 4 (ID4) Is Highly Expressed in Human Melanoma Tissues and May Function to Restrict Normal Differentiation of Melanoma Cells
Source: PLoS One. 2015 Feb 2;10(2):e0116839. doi: 10.1371/journal.pone.0116839 (PMC4314081; doi:10.1371/journal.pone.0116839)
Supplement: S1 Table — A total of 89 genes were upregulated. The fold-upregulation versus both the OM and NM samples is provided. The criteria used for inclusion in this list was that expression was at least 5-fold greater in MB samples than both the OM and NM samples (MB>OM, MB>NM). (DOC) [file pone.0116839.s005.doc]

**Table S1**

| **Accession#** | **Gene** | **Description** | **MB>OM** | **MB>NM** |
| --- | --- | --- | --- | --- |
| NM_015162 | ACSBG1 | acyl-CoA synthetase bubblegum family member 1 | 8.01 | 8.49 |
| NM_000681 | ADRA2A | adrenergic, alpha-2A-, receptor | 9.02 | 9.17 |
| NM_178563 | AGBL3 | TP/GTP binding protein-like 3 | 15.35 | 32.01 |
| NM_032785 | AGBL4 | ATP/GTP binding protein-like 4 | 60.94 | 36.3 |
| NM_139314 | ANGPTL4 | angiopoietin-like 4 | 908.28 | 94.67 |
| NM_000039 | APOA1 | apolipoprotein A-I | 25.47 | 13.99 |
| NM_000047 | ARSE | arylsulfatase E (chondrodysplasia punctata 1) | 5.80 | 6.42 |
| NM_024490 | ATP10A | ATPase, Class V, type 10A | 21.40 | 8.11 |
| NM_020632 | ATP6V0A4 | ATPase, H+ transporting, lysosomal V0 subunit a4 | 5.02 | 8.07 |
| NM_006576 | AVIL | advillin | 8.15 | 12.62 |
| NM_001713 | BHMT | betaine-homocysteine methyltransferase | 10.28 | 10.48 |
| NM_001202 | BMP4 | bone morphogenetic protein 4 | 8.85 | 6.52 |
| NM_133468 | BMPER | BMP binding endothelial regulator | 13.92 | 13.06 |
| NM_183393 | CADPS | Ca2+-dependent secretion activator | 8.73 | 6.34 |
| NM_001295 | CCR1 | chemokine (C-C motif) receptor 1 | 5.19 | 15.39 |
| NM_178445 | CCRL1 | chemokine (C-C motif) receptor-like 1 | 31.59 | 13.17 |
| NM_021153 | CDH19 | cadherin 19, type 2 | 16.34 | 6.01 |
| NM_004370 | COL12A1 | collagen, type XII, alpha 1 | 10.33 | 7.66 |
| NM_031311 | CPVL | carboxypeptidase, vitellogenic-like | 6.73 | 22.93 |
| NM_001012288 | CRLF2 | Cytokine receptor-like factor 2 | 36.01 | 31.18 |
| NM_018659 | CYTL1 | cytokine-like 1 | 5.85 | 8.03 |
| NM_004717 | DGKI | diacylglycerol kinase | 7.41 | 19.42 |
| NM_016246 | DHRS10 | dehydrogenase/reductase (SDR family) member 10 | 11.96 | 7.53 |
| NM_001937 | DPT | dermatopontin (DPT) | 28.74 | 19.97 |
| S58722 | DXS542 | X-linked retinopathy protein | 30.06 | 16.71 |
| NM_003991 | EDNRB | endothelin receptor type B | 13.32 | 8.49 |
| NM_003633 | ENC1 | ectodermal-neural cortex (with BTB-like domain) | 5.09 | 12.49 |
| NM_001982 | ERBB3 | Epidermal growth factor receptor | 6.62 | 12.55 |
| NM_000129 | F13A1 | coagulation factor XIII, A1 polypeptide | 25.07 | 17.42 |
| NM_178128 | FADS6 | fatty acid desaturase domain family, member 6 | 13.20 | 16.51 |
| NM_144594 | FAM112B | family with sequence similarity 112 | 21.40 | 12.74 |
| NM_017709 | FAM46C | family with sequence similarity 46 | 13.89 | 9.93 |
| NM_145032 | FBXL13 | F-box and leucine-rich repeat protein 13 | 5.35 | 5.82 |
| NM_023111 | FGFR1 | fibroblast growth factor receptor 1 | 5.52 | 13.87 |
| NM_013231 | FLRT2 | fibronectin leucine rich transmembrane protein 2 | 5.79 | 8.17 |
| NM_002023 | FMOD | fibromodulin | 14.15 | 17.70 |
| NM_001136011 | FXYD3 | FXYD-domain containing regulators of Na+/K+ ATPase | 26.27 | 18.17 |
| NM_001495 | GFRA2 | GDNF family receptor alpha 2 | 28.31 | 40.22 |
| NM_000166 | GJB1 | gap junction protein, beta 1 | 9.02 | 29.47 |
| NM_015234 | GPR116 | G protein-coupled receptor 116 | 8.53 | 7.11 |
| NM_032787 | GPR128 | G protein-coupled receptor 128 | 65.43 | 45.46 |
| NM_152529 | GPR155 | G protein-coupled receptor 155 | 5.65 | 7.37 |
| NM_000830 | GRIK1 | glutamate receptor, ionotropic, kainate 1 | 6.06 | 12.25 |
| NM_003520 | HIST1H2BN | histone 1, H2bn | 28.28 | 15.72 |
| NM_031935 | HMCN1 | hemicentin 1 | 5.50 | 7.11 |
| NM_002130 | HMGCS1 | 3-hydroxy-3-methylglutaryl-Coenzyme A synthase 1 (soluble) | 8.91 | 6.94 |
| NM_001546 | ID4 | inhibitor of DNA binding 4, dominant negative helix-loop-helix protein (ID4) | 21.64 | 13.98 |
| NM_001557 | IL8RB | interleukin 8 receptor | 56.86 | 33.87 |
| NM_003637 | ITGA10 | integrin, alpha 10 | 8.45 | 7.98 |
| NM_001024660 | KALRN | kalirin, RhoGEF kinase (KALRN), transcript variant 1 | 5.57 | 6.03 |
| NM_198439 | KBTBD3 | kelch repeat and BTB | 75.21 | 52.26 |
| NM_004976 | KCNC1 | potassium voltage-gated channel, Shaw-related subfamily, member 1 | 21.25 | 7.29 |
| NM_000226 | KRT9 | type I keratin 9 | 5.08 | 8.75 |
| NM_014240 | LIMD1 | LIM domains containing 1 | 5.8 | 7.62 |
| NM_005824 | LRRC17 | leucine rich repeat containing 17 | 5.33 | 27.50 |
| AJ312775 | LUZPP1 | leucine zipper protein 3 | 119.24 | 71.02 |
| NM_139021 | MAPK15 | mitogen-activated protein kinase 15 | 25.27 | 12.39 |
| NM_024979 | MCF2L | MCF.2 cell line derived transforming sequence-like | 56.15 | 24.36 |
| NM_000900 | MGP | matrix Gla protein | 45.60 | 79.67 |
| NM_014429 | MORC1 | MORC family CW-type zinc finger 1 | 10.45 | 7.95 |
| NM_138983 | OLIG1 | oligodendrocyte transcription factor 1 | 5.59 | 6.43 |
| NM_145260 | OSR1 | odd-skipped related 1 | 7.59 | 18.09 |
| NM_173582 | PGM2L1 | phosphoglucomutase 2-like 1 | 8.28 | 7.80 |
| NM_015553 | PIP3-E | phosphoinositide-binding protein PIP3-E | 53.60 | 37.25 |
| XM_007622012 | PLA1A | phospholipase A1 member A | 7.11 | 8.81 |
| NM_025179 | PLXNA2 | plexin-A family of semaphorin co-  receptors | 10.9 | 16.34 |
| NM_002725 | PRELP | proline/arginine-rich end leucine-rich repeat protein | 17.65 | 6.05 |
| NM_000264 | PTCH | patched homolog | 6.51 | 11.82 |
| NM_000962 | PTGS1 | prostaglandin-endoperoxide synthase 1 | 37.7 | 20.96 |
| NM_002864 | PZP | pregnancy-zone protein, proteinase inhibitor | 36.18 | 10.11 |
| NM_016563 | RASL12 | RAS-like, family 12 | 10.26 | 7.19 |
| NM_002903 | RCV1 | recoverin | 22.01 | 15.29 |
| NM_000326 | RLBP1 | retinaldehyde binding protein 1 | 11.15 | 20.58 |
| NM_031916 | ROPN1L | ropporin 1-like | 13.01 | 9.93 |
| NM_006917 | RXRG | retinoid X receptor gamma | 39.57 | 16.59 |
| NM_002961 | S100A4 | S100 calcium binding protein A4 | 32.77 | 32.14 |
| NM_001043 | SLC6A2 | solute carrier family 6 (neurotransmitter transporter, noradrenalin) , member 2 | 25.27 | 15.05 |
| NM_144775 | SMCR8 | Smith-Magenis syndrome chromosome region | 11.77 | 9.03 |
| NM_003106 | SOX2 | SRY (sex determining region Y)-box 2 | 14.53 | 24.18 |
| NM_030965 | ST6GALNAC5 | ST6 (alpha-N-acetyl-neuraminyl-2,3-beta-galactosyl-1,3)-N-acetylgalactosaminide alpha-2,6-sialyltransferase 5 | 16.73 | 6.97 |
| AB028977 | SV2C | mRNA for KIAA1054 protein | 24.46 | 16.99 |
| NM_138810 | TAGAP | T-cell activation GTPase activating protein | 25.88 | 17.98 |
| NM_003273 | TM7SF2 | transmembrane 7 superfamily member 2 | 5.91 | 7.64 |
| NM_030788 | TM7SF4 | transmembrane 7 superfamily member 4 | 6.2 | 8.32 |
| NM_003284 | TNP1 | transition protein 1 (during histone to protamine replacement) | 58.60 | 32.57 |
| NM_147204 | TRPV4 | transient receptor potential cation channel, subfamily V, member 4 | 5.15 | 6.19 |
| NM_152275 | TTC30A | tetratricopeptide repeat domain 30A | 6.96 | 9.95 |
| NM_053276 | VIT | vitrin | 6.80 | 7.30 |
| NM_152520 | ZNF533 | zinc finger protein 533 | 32.86 | 18.27 |
