# Supplementary material for: Inhibitor of DNA Binding 4 (ID4) Is Highly Expressed in Human Melanoma Tissues and May Function to Restrict Normal Differentiation of Melanoma Cells
Source: PLoS One. 2015 Feb 2;10(2):e0116839. doi: 10.1371/journal.pone.0116839 (PMC4314081; doi:10.1371/journal.pone.0116839)
Supplement: S2 Table — A total of 41 genes were downregulated. The fold-downregulation versus both the OM and NM samples is provided. The criteria used for inclusion in this list was that expression was at least 5-fold lower in MB samples than both the OM and NM samples (MB<OM, MB<NM). (DOC) [file pone.0116839.s006.doc]

**Table S2**

| **Accession#** | **Gene** | **Description** | **MB<OM** | **MB<NM** |
| --- | --- | --- | --- | --- |
| NM_005502 | ABCA1 | ATP-binding cassette transporter | 74.57 | 98.13 |
| NM_174858 | AK5 | adenylate kinase 5 | 8.87 | 7.69 |
| NM_153228 | ANKFN1 | ankyrin-repeat and fibronectin type III | 14.33 | 50.51 |
| NM_004024 | ATF3 | activating transcription factor 3 | 5.41 | 10.46 |
| NM_138553 | BCL11A | B-cell CLL/lymphoma 11A | 10.36 | 14.43 |
| NM_024111 | CHAC1 | cation transport regulator-like 1 | 14.3 | 18.03 |
| NM_004895 | CIAS1 | cold autoinflammatory syndrome 1 | 5.63 | 12.73 |
| NM_005203 | COL13A1 | collagen, type XIII, alpha 1 | 9.55 | 9.67 |
| NM_001511 | CXCL1 | chemokine (C-X-C motif) ligand 1 | 5.88 | 26.50 |
| NM_004887 | CXCL14 | chemokine (C-X-C motif) ligand 14 | 19.55 | 18.38 |
| NM_002089 | CXCL2 | chemokine (C-X-C motif) ligand 2 | 5.18 | 30.55 |
| NM_002090 | CXCL3 | chemokine (C-X-C motif) ligand 3 | 11.62 | 45.61 |
| NM_001554 | CYR61 | cysteine-rich, angiogenic inducer, 61 | 12.06 | 9.67 |
| NM_019058 | DDIT4 | DNA-damage-inducible transcript 4 | 5.85 | 9.84 |
| NM_012242 | DKK1 | dickkopf homolog 1 | 6.27 | 6.65 |
| NM_001393 | ECM2 | extracellular matrix protein 2 | 6.58 | 17.05 |
| NM_144503 | F11R | F11 receptor (F11R) | 13.21 | 10.22 |
| NM_030797 | FAM49A | family with sequence similarity 49, member A | 12.62 | 39.47 |
| NM_000148 | FUT1 | fucosyltransferase 1 (galactoside 2-alpha-L-fucosyltransferase | 31.5 | 364.7 |
| NM_000853 | GSTT1 | glutathione S-transferase theta 1 | 6.71 | 26.57 |
| NM_002141 | HOXA4 | homeobox A4 | 5.79 | 6.72 |
| NM_000575 | IL1A | interleukin 1, alpha | 56.16 | 22.03 |
| NM_000576 | IL1B | interleukin 1, beta (IL1B) | 5.54 | 9.48 |
| NM_000600 | IL6 | interleukin 6 (interferon, beta 2) | 9.08 | 16.25 |
| NM_000584 | IL8 | interleukin 8 | 14.9 | 88.31 |
| NM_000237 | LPL | lipoprotein lipase | 7.68 | 9.50 |
| NM_007161 | LST1 | leukocyte specific transcript 1 | 9.32 | 8.61 |
| NM_002421 | MMP1 | matrix metallopeptidase 1 | 11.89 | 9.53 |
| NM_004994 | MMP9 | matrix metallopeptidase 9 | 6.72 | 13.70 |
| NM_024761 | MOBKL2B | Mps One Binder kinase activator-like 2B | 8.94 | 26.93 |
| NM_033120 | NKD2 | naked cuticle homolog 2 | 5.14 | 7.45 |
| NM_004563 | PCK2 | phosphoenolpyruvate carboxykinase 2 | 5.62 | 10.81 |
| NM_058179 | PSAT1 | phosphoserine aminotransferase 1 | 6.22 | 14.33 |
| NM_002852 | PTX3 | pentraxin-related gene, rapidly induced by IL-1 beta | 7.92 | 8.66 |
| NM_171998 | RAB39B | member RAS oncogene family | 30.15 | 60.98 |
| NM_002888 | RARRES1 | retinoic acid receptor responder | 5.14 | 10.1 |
| NM_003835 | RGS9 | regulator of G-protein signalling 9 | 6.91 | 15.02 |
| NM_144569 | SPOCD1 | SPOC domain containing 1 | 7.54 | 6.22 |
| NM_003714 | STC2 | stanniocalcin 2 | 13.03 | 25.19 |
| NM_014848 | SV2B | synaptic vesicle glycoprotein 2B | 13.03 | 37.85 |
| NM_001561 | TNFRSF9 | tumor necrosis factor receptor | 10.59 | 76.1 |
