# Supplementary material for: Inhibitor of DNA Binding 4 (ID4) Is Highly Expressed in Human Melanoma Tissues and May Function to Restrict Normal Differentiation of Melanoma Cells
Source: PLoS One. 2015 Feb 2;10(2):e0116839. doi: 10.1371/journal.pone.0116839 (PMC4314081; doi:10.1371/journal.pone.0116839)
Supplement: S1 Methods — (DOC) [file pone.0116839.s007.doc]

**Methods S1. Materials And Methods for experiments in Figures S1 and S3.**

**Cell growth assay.**

Dye-labeling was used to monitor cell growth over time under standard and hESC growth conditions. Dye retention was measured using the Guava EasyCyte instrument (EMD Millipore, Billerica, MA). Cells (5 X 10^4^) were plated in 24-well dishes overnight. After a PBS wash, cells were pulsed for 15 min using the Carboxyfluorescein Succinimidyl Ester (CFSE) reagent component of the Guava Cell Growth Kit for Flow Cytometry (cat. no. 4500-0270, EMD Millipore, Billerica, MA), at a final concentration of 2.5μM in PBS. Cells were washed with RPMI-1640 media (without serum) and then propagated in standard (RPMI-1640 medium, 10% FCS) or hESC culture media with replenishment every 24 hours. Cell samples were analyzed at different time points to quantitate dye retention.

**Cell cycle assay.**

Cell cycle assays were performed using Guava Cell Cycle Reagent for Flow Cytometry (cat. no. 4500-0220, EMD Millipore, Billerica, MA) according to the manufacturer protocol. Briefly, cells were plated in 96 well dishes (2.5 X 10^4^ cells per well) and left to adhere overnight. Cells were trypsinized, collected by centrifugation, and fixed in 70% ethanol overnight at -20 °C. Cells were then washed with PBS and stained with Guava Cell Cycle Reagent for 30 min at room temperature. Samples were read and analyzed using the Guava EasyCyte instrument.
